# Reducing translation through eIF4G/IFG‐1 improves survival under ER stress that depends on heat shock factor HSF‐1 in *Caenorhabditis elegans*

**DOI:** 10.1111/acel.12516

**Published:** 2016-08-18

**Authors:** Amber C. Howard, Jarod Rollins, Santina Snow, Sarah Castor, Aric N. Rogers

**Affiliations:** ^1^ MDI Biological LaboratoryDavis Center for Regenerative Biology and Medicine159 Old Bar Harbor RoadSalisbury CoveME04672USA; ^2^ The Jackson Laboratory600 Main StreetBar HarborME04609USA; ^3^ Present address: Natural Lab ScienceUniversity of Maine at AugustaJH 102CAugustaME04330USA

**Keywords:** *Caenorhabditis elegans*, eIF4G, healthspan, *ifg‐1*, lifespan, proteostasis

## Abstract

Although certain methods of lowering and/or altering mRNA translation are associated with increased lifespan, the mechanisms underlying this effect remain largely unknown. We previously showed that the increased lifespan conferred by reducing expression of eukaryotic translation initiation factor 4G (eIF4G/IFG‐1) enhances survival under starvation conditions while shifting protein expression toward factors involved with maintaining ER‐dependent protein and lipid balance. In this study, we investigated changes in ER homeostasis and found that lower eIF4G/IFG‐1 increased survival under conditions of ER stress. Enhanced survival required the ER stress sensor gene *ire‐1* and the ER calcium ATPase gene *sca‐1* and corresponded with increased translation of chaperones that mediate the ER unfolded protein response (UPR^ER^). Surprisingly, the heat‐shock transcription factor gene *hsf‐1* was also required for enhanced survival, despite having little or no influence on the ability of wild‐type animals to survive ER stress. The requirement for *hsf‐1* led us to re‐evaluate the role of eIF4G/IFG‐1 on thermotolerance. Results show that lowering expression of this translation factor enhanced thermotolerance, but only after prolonged attenuation, the timing of which corresponded to increased transcription of heat‐shock factor transcriptional targets. Results indicate that restricting overall translation through eIF4G/IFG‐1 enhances ER and cytoplasmic proteostasis through a mechanism that relies heavily on *hsf‐1*.

## Introduction

Regulation of protein synthesis is essential during development and in response to environmental inputs. Proper regulation insures that cells have the resources required for maintaining cell structure and function. Excess synthesis or an inability to turn down production during times of stress may cause proteotoxicity due to the inability of the cell to properly fold, refold, and degrade what is manufactured (Sherman & Qian, 2013). Thus, numerous controls are in place at different stages of protein synthesis to insure a proper balance.

In the last decade, a number of studies determined that genetic suppression of translation results in increased lifespan in model organisms including yeast, worms, flies, and mammals (Kaeberlein & Kennedy, 2011; Kyriakakis *et al*., 2015). Translation is a cellular process with inputs from major longevity‐regulating pathways including the target of rapamycin (TOR) and insulin/IGF‐1 pathways. TOR integrates intracellular energy and nutrient signals to coordinate multiple cellular processes, including translation (Kapahi *et al*., 2010). The insulin/IGF‐1 pathway coordinates development as well as challenges to homeostasis through intercellular hormone signaling and the FOXO transcription factor DAF‐16 (abnormal dauer formation; Kenyon, 2010). Inhibition of the insulin/IGF‐1 pathway in *Caenorhabditis elegans* decreases ribosomal subunits resulting in reduced protein synthesis (Depuydt *et al*., 2013). In addition to genetic models of lifespan extension, dietary restriction also induces translation inhibition. In fact, reducing translation by limiting dietary intake of the essential amino acid methionine, but not total calories, extends lifespan (Miller *et al*., 2005). While translation inhibition may not be essential for every genetic or environmental model of lifespan extension, the frequency with which it is involved in longevity models and the rigor of the response across species tested suggests a has led to the proposal that translation plays a universal role in the aging process (Tavernarakis, 2008).

A possible explanation for why attenuating translation increases lifespan is enhanced proteostasis due to improved translation fidelity and/or turnover. Recent support for part of this idea was demonstrated by a study which showed that slowing down translation was sufficient to increase fidelity of protein folding, even among mutant proteins with a propensity for misfolding (Meriin *et al*., 2012). On the other hand, other studies have shown that a shift in mRNA translation preference (i.e., differential translation) accompanying an overall reduction of protein synthesis is a key component to increased lifespan in yeast (Steffen *et al*., 2008), *C. elegans* (Rogers *et al*., 2011), and *Drosophila* (Zid *et al*., 2009). Differential mRNA translation has long been known to be important in response to cellular stressors (Holcik & Sonenberg, 2005). Additional clues to the mechanisms underlying longevity effects are likely to come from a better understanding of associated cellular changes, which may be dependent on the manner in which translation is modulated and which require an understanding of its major regulators.

Although controlled at multiple levels, translation begins with, and is most frequently rate‐limited by, the initiation stage (Hershey *et al*., 2012). A major determinant of regulation at this stage is the cap‐binding complex, which is made up of the eukaryotic translation initiation factor (eIF)4G, the eIF4A RNA helicase, and the 5′ mRNA cap‐binding protein eIF4E. eIF4G acts as a nexus for translation by bringing in other translation factors and helping recruit the small (40S) ribosomal subunit. Although eIF4E or eIF4G can become limiting for cap‐mediated translation, only eIF4G is also involved in cap‐independent translation and has been shown to be a limiting factor in yeast and worms under nutrient stress or when TOR signaling is attenuated (Berset *et al*., 1998; Ramirez‐Valle *et al*., 2008; Rogers *et al*., 2011). While attenuating expression of eIF4G slows growth and negatively impacts fertility (Pan *et al*., 2007; Contreras *et al*., 2011), it extends lifespan in yeast (Smith *et al*., 2008) and *C. elegans* (Curran & Ruvkun, 2007; Hansen *et al*., 2007; Pan *et al*., 2007).

To help explain the longevity phenotype, we previously showed that reducing *ifg‐1*, the eIF4G homolog in *C. elegans*, switched gene‐specific translation from a pro‐growth mode to one that is pro‐survival (Rogers *et al*., 2011). Analysis revealed differential translation that enhanced relative expression of genes important for responding to stress and positive regulators of lifespan (Rogers *et al*., 2011). In other systems, overexpression of this translation factor is associated with disease pathogenesis. For example, overexpression of eIF4G in mammalian cells is sufficient to induce transformation (Fukuchi‐Shimogori *et al*., 1997), while in humans, overexpression of eIF4G is associated with breast, pharyngeal, and lung cancer (Bauer *et al*., 2002; Cromer *et al*., 2003; Braunstein *et al*., 2007). Opposing effects of eIF4G inhibition and overexpression on longevity and disease, along with its impact on the translatome, led us to investigate the intracellular processes downstream of translational remodeling that are important for maintaining organismal homeostasis.

Taking genes found to be differentially translated when eIF4G/IFG‐1 is reduced, we performed an ontological analysis of cellular components and found that protein synthesis of factors that are part of vesicle trafficking and the endomembrane system are promoted. This system involves factors that are manufactured by, and participate in endoplasmic reticulum (ER)‐associated processes. The ER is a site of protein synthesis, which can become perturbed in response to misfolded proteins and/or ER calcium imbalance. Stress in this organelle induces the unfolded protein response (UPR^ER^), which was previously shown to regulate longevity in a cell‐nonautonomous manner in *C. elegans* (Taylor & Dillin, 2013). The UPR^ER^ is guided by ER stress sensors IRE‐1 (inositol‐requiring enzyme 1), PERK/PEK‐1 (protein kinase RNA‐like ER kinase 1) and ATF‐6 (activation transcription factor 6). Thus, we tested the impact of reducing *ifg‐1* under ER stress conditions initiated by tunicamycin, which blocks glycosylation and activates the UPR^ER^. Results showed improved survival in a manner dependent on the gene encoding an ER calcium ATPase, *sca‐1*. This gene is essential for ER homeostasis in *C. elegans* (Yan *et al*., 2006) and was previously shown to be required for extended lifespan when *ifg‐1* is reduced (Rogers *et al*., 2011).

Additionally, we tested the known sensors of ER stress to determine their requirement for enhanced survival when *ifg‐1* is attenuated. We found that increased protection under ER stress conditions was dependent on the IRE‐1 branch of the UPR^ER^. Neither ATF‐6 nor the translation inhibiting kinase PEK‐1 was required for increased ER stress tolerance when *ifg‐1* was reduced. Surprisingly, we also found a requirement for the heat‐shock factor HSF‐1 in enhanced ER stress tolerance, a transcription factor that regulates the cytoplasmic heat‐shock response (HSR). Tests for resistance to thermal stress showed that RNAi targeting *ifg‐1* required several days of suppression, despite strongly reduced IFG‐1 protein levels after only 2 days. Interestingly, the delayed protective response was accompanied by increased constitutive expression of HSF‐1 targets. Enhanced constitutive expression of heat‐shock factor gene expression, including the temporal delay, was only observed for certain translational interventions tested. From a focused prospective, results indicate that changes in the cytoplasmic HSR are critical to enhanced ER function when translation is modulated by reducing *ifg‐1*. From a broader perspective, findings from tests using multiple methods of translation attenuation suggest a new basis for discerning translation‐mediated pathways of stress resistance.

## Results

### Restricting translation by lowering *ifg‐1* improved survival under ER stress

**Figure 1:**
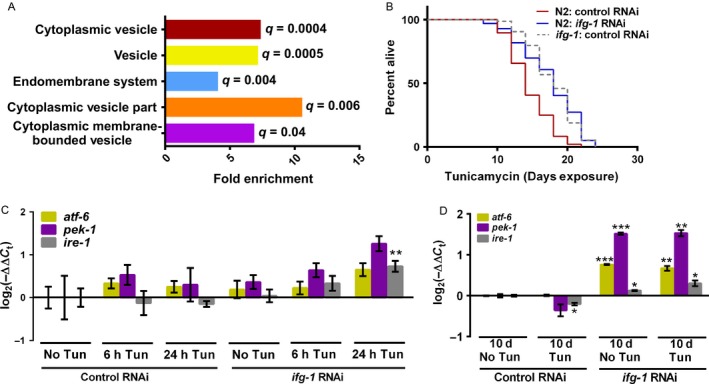
Reduced translation through *ifg‐1* promoted survival under ER stress. (A) Cellular component GO analysis of differentially translated genes associated with suppressing *ifg‐1* (*q* = false discovery rate). Additional information is available in the Experimental procedures section. (B) Survival of N2 wild‐type animals fed bacteria expressing control (L4440) or *ifg‐1* dsRNA starting on the first day of adulthood, followed by exposure to 25 μg mL^−1^ tunicamycin 2 days later. *ifg‐1*(*cxTi9279*) mutants exposed to tunicamycin were included in the survival assay for comparison. Experiments were performed four or more times with similar results (see Table S2, Supporting information for additional data). Kaplan–Meier survival curves were compared using Mantel–Cox log‐rank test (*P* < 0.0001 for N2 survival on control vs. *ifg‐1* RNAi; *P* < 0.0001 for N2 and *ifg‐1*(*cxTi9279*) on control RNAi; *P* = 0.94 for N2 on *ifg‐1* RNAi vs. *ifg‐1*(*cxTI9279*) animals on control RNAi). (C) N2 wild‐type animals were fed control or *ifg‐1* dsRNA for 4 days to allow for full effect of RNAi prior to exposure to 25 μg mL^−1^ tunicamycin. Relative mRNA levels of *atf‐6*,* pek‐1*, and *ire‐1* are shown after six or 24 h of exposure (6 h Tun and 24 h Tun, respectively). Samples were normalized to similarly prepared animals exposed to DMSO for 6 h (No Tun). Results are from three separate experiments (**P < 0.001; two‐tailed unpaired t‐test; error bars represent SEM). (D) N2 adults treated with either control or *ifg‐1* RNAi starting at adulthood were exposed to 25 μg mL^−1^ tunicamycin from day 4 to day 10 of adulthood (10 days Tun). Relative mRNA levels of *atf‐6*,* pek‐1*, and *ire‐1* were measured at day 10. Samples were normalized to animals exposed to DMSO over the same period. Results were from three separate experiments (*P < 0.05, **P < 0.001, ***P < 0.0001, two‐tailed unpaired *t*‐test; error bars represent SEM). In all experiments, results are considered significant for *P* < 0.05.

Previous analysis showed that increased lifespan resulting from reduced *ifg‐1* expression in *C. elegans* was at least partially dependent on several translationally upregulated genes (Rogers *et al*., 2011). Those most important for increased lifespan are also known to be important for maintaining ER homeostasis and include the ER calcium regulator gene *sca‐1* and a gene encoding the transcriptional coactivator mediator complex subunit *mdt‐15*. Attenuated expression of either is able to induce the UPR^ER^ (Yan *et al*., 2006; Hou *et al*., 2014b). A novel cellular component‐based gene ontological (GO) analysis of genes that were differentially translated when *ifg‐1* is reduced via RNAi (Rogers *et al*., 2011) revealed that cellular processes involving vesicle transport and the endomembrane system were significantly overrepresented (Fig. 1A; Table S1, Supporting information). Furthermore, 75% of the differentially translated genes in these GO categories had increased translation according to changes in the association of mRNA with ribosomes. Given the role of the ER in protein synthesis and maintenance of proteostasis, we investigated the effect of reduced *ifg‐1* expression under conditions that induce the UPR^ER^.

Animals were fed double‐stranded (ds)RNA corresponding to *ifg‐1* or a control vector starting at adulthood followed by exposure to the glycosylation‐inhibiting drug tunicamycin. Survival was significantly improved in animals with *ifg‐1* RNAi, with protective effects also observed in mutants bearing the *ifg‐1* loss‐of‐function allele *cxTi9279* (Fig. 1B; Table S2, Supporting information). Baseline expressions of ER stress sensor genes *ire‐1*,* atf‐6*, and *pek‐1* were unchanged in *ifg‐1* RNAi‐treated animals (Fig. 1C) and *ifg‐1*(*cxTi9279*) mutants in the absence of stress (Fig. S1A, Supporting information). However, in animals subjected to *ifg‐1* RNAi, a small but significant increase in *ire‐1* transcript levels was observed in response to 24 h of exposure to tunicamycin (Fig. 1C), whereas the *ifg‐1* mutant showed only a small increase in *pek‐1* and decrease in *atf‐6* under the same conditions (Fig. S1A, Supporting information). Thus, lowering *ifg‐1* increased survival in response to ER stress without major changes in ER stress sensor transcript levels compared with RNAi control animals in a 24‐h window after treatment with tunicamycin.

To see whether knockdown of *ifg‐1* changed expression of ER stress sensors with time, measurements were made at day 10 of adulthood, which is closer to the time that a difference in survival was observed between test and control animals. Interestingly, a small but significant increase was observed for each sensor in *ifg‐1* RNAi‐treated animals regardless of whether they were exposed to tunicamycin, with a greater than twofold change in *pek‐1* by this time (Fig. 1D). These changes were consistent whether a 2‐day (Fig. S1B, Supporting information) or 4‐day (Fig. 1D) RNAi induction phase was used prior to tunicamycin exposure. These results indicated that lowering translation through *ifg‐1* resulted in long‐term enhanced expression of ER stress sensors even in the absence of external stress.

**Figure 2:**
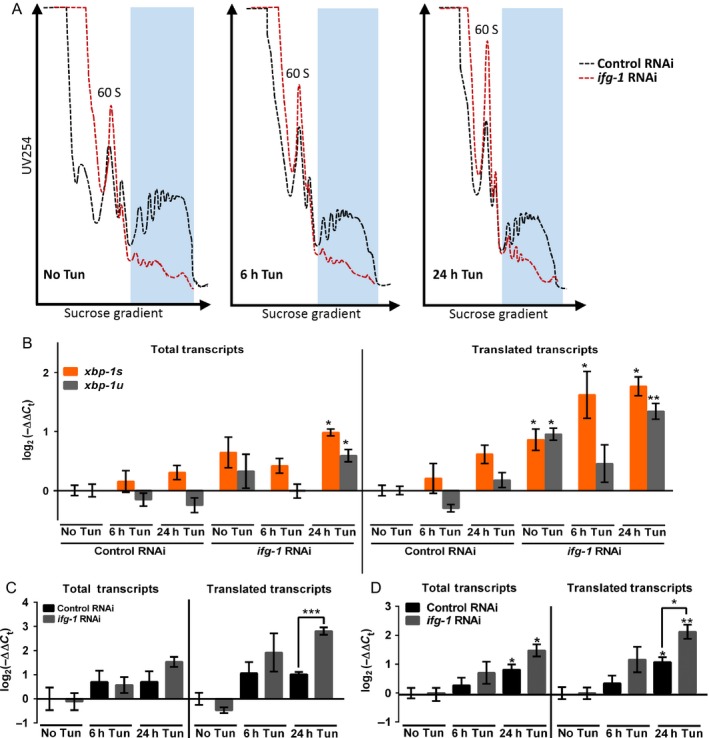
Reducing expression of *ifg‐1* promoted transcriptional and translational upregulation of UPR^ER^‐responsive genes under ER stress. (A) Polysome profiles for wild‐type N2 adults fed bacteria expressing L4440 control (black dashes) or *ifg‐1* dsRNA (red dashes) for 4 days prior to exposure to 25 μg mL^−1^ tunicamycin for 6 and 24 h (6 h Tun and 24 h Tun, respectively). Nematode lysates were separated over a sucrose gradient and absorbance through the gradient was measured at 254 nm (UV254). Portions of the profile corresponding to polysomes are highlighted in blue and were collected from each sample for analysis of translated mRNA. Profiles are representative of four experiments performed. (B) Total and translated (polysome‐associated) mRNA levels of *xbp‐1s* and *xbp‐1u* were measured under conditions in (A) (*P < 0.05, **P < 0.001; two‐tailed *t*‐test; error bars indicate SEM). (C) Total and translated mRNA levels for *hsp‐4* were measured under conditions in (A) (***P < 0.0001; two‐tailed *t*‐test; error bars indicate SEM). (D) Similar to (C), but for *dnj‐7* (*P < 0.05, **P < 0.001; two‐tailed *t*‐test; error bars indicate SEM). Results in A–D are from the same four biological replicates and were considered significant for *P* < 0.05.

As ER stress sensor activity can induce both transcriptional and translational effects, we used polysome profiling to assess changes in global and differential translation. Upon reaching adulthood, animals were fed dsRNA for 4 days prior to profiling. *ifg‐1* RNAi dramatically decreased total translation and increased free ribosomal subunits compared with controls (Fig. 2A, left panel). An increase in free ribosomal subunits was observed in both control and *ifg‐1* RNAi animals 6 h after treatment with tunicamycin that continued to increase by 24 h (Fig. 2A, middle and right panels). This indicated that global translation was reduced throughout this period in response to tunicamycin for animals under control or *ifg‐1* RNAi. Indeed, attenuation of translation is a conserved response to induction of the UPR^ER^ (Ron & Walter, 2007).

To see whether translation suppression in response to tunicamycin was related to the PEK‐1/PERK arm of the UPR^ER^, we examined the inhibitory phosphorylation status of its protein target, eIF2α, which is part of the mRNA translation ternary complex and represents a separate (*ifg‐1‐*independent) rate‐limiting regulator of translation initiation (Holcik & Sonenberg, 2005). eIF2α phosphorylation was lower in animals treated with *ifg‐1* RNAi than control RNAi prior to tunicamycin exposure (Fig. S2A, Supporting information). Subsequently, both control and *ifg‐1* RNAi‐treated animals showed an upward tendency of eIF2α phosphorylation after 6 h of exposure to tunicamycin that disappeared by 24 h (Fig. S2A, Supporting information). No significant differences in phosphorylation status between *ifg‐1* and control RNAi animals were observed at six and 24 h. Although a tendency for increased eIF2α phosphorylation after 6 h of tunicamycin treatment are in line with the modest translational reduction in *ifg‐1* and control RNAi‐treated animals observed in polysome profiles, the continued abatement of translation after 24 h in the absence of increased eIF2α phosphorylation suggested that translation may be attenuated by additional factors.

To determine the response of the IRE‐1 branch of the UPR^ER^, we measured transcription and translation of its target, *xbp‐1* mRNA. In its active state, IRE‐1 splices immature *xbp‐1u* (unspliced) transcripts, which allows translation of the mature *xbp‐1s* (spliced) transcript and synthesis of the transcription factor that it encodes (Ron & Walter, 2007). Prior to tunicamycin treatment, total transcript expression of *xbp‐1u* and *xbp‐1s* were comparable between control and *ifg‐1* RNAi‐treated animals (Fig. 2B, left panel). Interestingly, recruitment of *xbp‐1s* and even the nontranslationally productive *xbp‐1u* to polysomes was constitutively higher in *ifg‐1* RNAi‐treated animals (Fig. 2B, right panel). The ratio of spliced to unspliced was similar prior to stress. Upon exposure to tunicamycin, there was a downward trend in total and translated transcripts of *xbp‐1u* and corresponding increase in *xbp‐1s* for all conditions (Fig. 2B), indicating activation of IRE‐1. The bias in expression of *xbp‐1s* abated somewhat by 24 h of tunicamycin treatment, but both total and translated forms of *xbp‐1s* increased in *ifg‐1* RNAi animals by this time. Despite starting at a higher constitutive (no tunicamycin) level, polysome‐associated *xbp‐1s* in *ifg‐1* RNAi animals increased relative to starting levels by 24 h of tunicamycin exposure (*P* = 0.0092).

Gene expression of several chaperones and other factors are dependent on the IRE‐1/XBP‐1 arm of the UPR^ER^. Expression of *hsp‐4*, which is orthologous to the mammalian binding immunoglobulin protein (BiP), is known to be highly dependent on activity of XBP‐1. We measured *hsp‐4* in *ifg‐1* RNAi‐treated animals and observed that its transcripts were more highly associated with polysomes after 24 h of tunicamycin treatment than in control RNAi animals (Fig. 2C, right panel). We also tested the DnaJ domain 7 gene, *dnj‐7*, which encodes the *C. elegans* ortholog of p58^IPK^, a peripheral ER protein that functions in ER associated degradation (ERAD) of misfolded proteins (Oyadomari *et al*., 2006). Analysis showed that tunicamycin induced *dnj‐7* transcription after 24 h of tunicamycin exposure under both conditions. However, animals with reduced *ifg‐1* showed a higher increase in polysome‐associated *dnj‐7* (Fig. 2D), suggesting that ERAD may be induced to a greater extent by the 24‐h time point. Recently, the protein disulfide isomerase family A member 6 (PDI‐6) was shown to control a negative feedback to the ER stress response by binding and inactivating IRE‐1 in *C. elegans* (Eletto *et al*., 2014). Expression analysis here indicated that, although total transcript levels of *pdi‐6* rose equally in response to control and *ifg‐1* RNAi after 24 h of tunicamycin exposure, reducing *ifg‐1* significantly increased *pdi‐6* polysome association (Fig. S2B, Supporting information). Such differences in UPR^ER^ activation could change the kinetics of the ER stress response and lead to faster resolution and rebalancing of organellar proteostasis. Resolution of the response would be indicated by a return of IRE‐1 activity to basal levels, and a tendency for this was observed between the six‐ and 24‐h time points in Fig. 2B by partial reversal of spliced vs. unspliced *xbp‐1* for animals under *ifg‐1* RNAi (especially among polysomes; Fig. 2B, right panel).

Genes encoding the sarco‐ER calcium ATPase SCA‐1 and the transcriptional coactivator subunit MDT‐15 are translationally upregulated and important for increased longevity when *ifg‐1* is attenuated (Rogers *et al*., 2011). Their requirement for maintaining ER homeostasis (Yan *et al*., 2006; Hou *et al*., 2014b) formed part of the rationale for investigating ER responses when translation is attenuated. However, changes in their translational status during the UPR^ER^ were unknown. Polysome association of *sca‐1* and *mdt‐15* transcripts increased when *ifg‐1* was reduced via RNAi (Fig. S2C,D, Supporting information). When exposed to ER stress from tunicamycin, attenuating *ifg‐1* promoted their translation but not their transcription, with no transcriptional nor translational effects in tunicamycin‐treated animals under control RNAi conditions (Fig. S2C,D, Supporting information). Results indicated that these genes were preferentially upregulated to the translation apparatus during the UPR^ER^ when translation was reduced through attenuation of *ifg‐1*. Taken together with their importance for increased lifespan when translation is attenuated, findings suggested that they may be important for increased survival under ER stress.

### Increased survival under ER stress was dependent on known regulators of proteostasis

**Figure 3:**
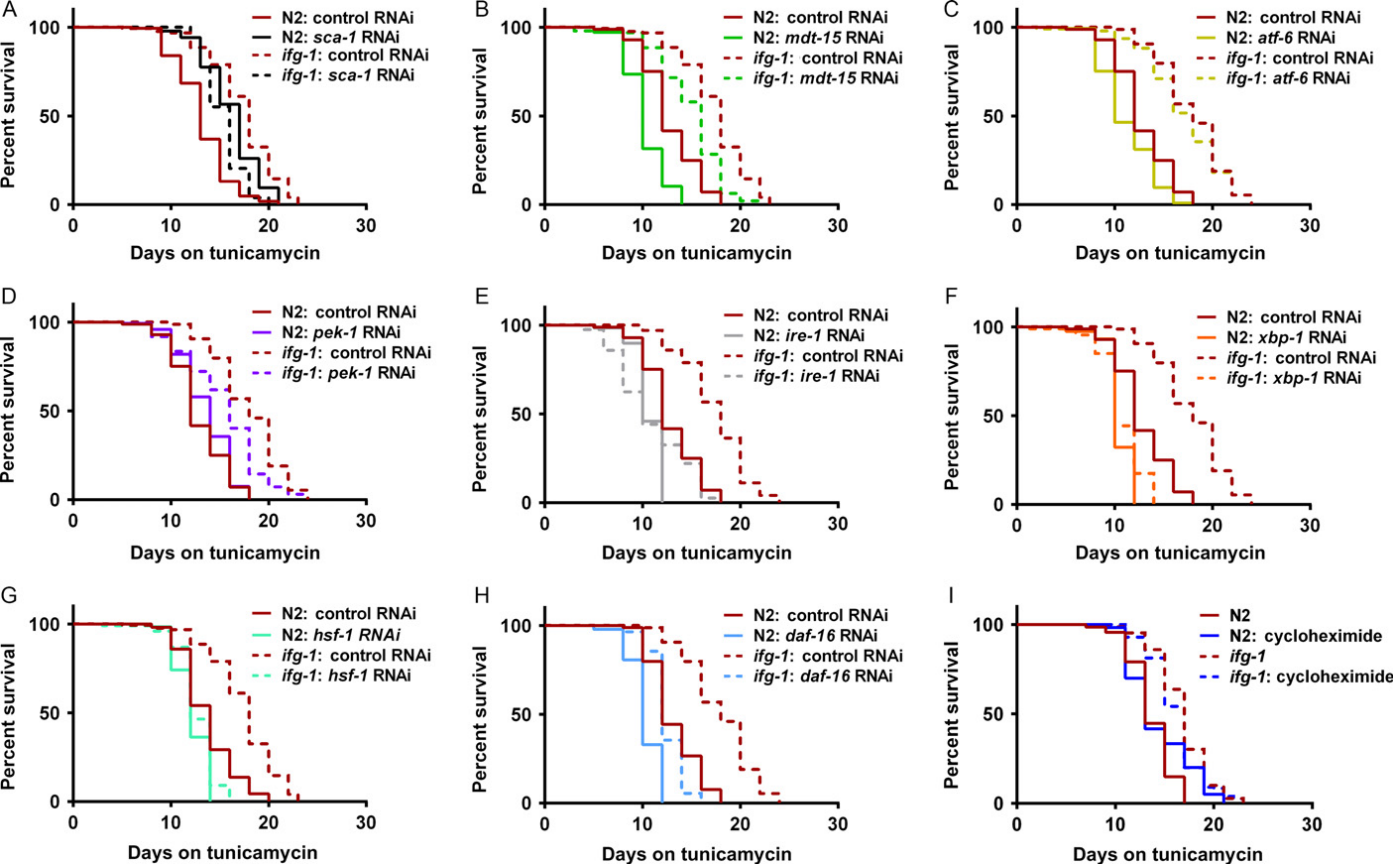
Enhanced survival in the *ifg‐1* mutant required *sca‐1*, the *ire‐1* branch of the UPR^ER^, and the cytoplasmic UPR gene *hsf‐1*. Day 1 adults were subjected to RNAi as indicated for 2 days prior to exposure to 25 μg mL^−1^ tunicamycin and survival was tracked daily. Kaplan–Meier survival curves were compared using Mantel–Cox log‐rank test, and average median lifespan for replicates was calculated using two‐tailed t‐test with Welch's correction. (A) The survival curve of *ifg‐1*(*cxTi9279*) fell below that of N2 when each were subjected to *sca‐1* RNAi (*P* < 0.0001). In (B–D), survival was enhanced in *ifg‐1*(*cxTi9279*) compared to N2 at *P* < 0.0001 for RNAi test conditions shown. In (E–G), average median survival was not enhanced in *ifg‐1*(*cxTi9279*) compared to N2 when each were subjected to *ire‐1* RNAi (*P* = 0.37), *xbp‐1* RNAi (*P* > 0.99), and *hsf‐1* RNAi (*P* = 0.18). (H) Survival was enhanced in *ifg‐1*(*cxTi9279*) compared to N2 when both were subjected to *daf‐16* RNA (*P* < 0.0001). (I) Survival was enhanced in *ifg‐1*(*cxTi9279*) compared to N2 when day 1 adults were pretreated with cycloheximide for 2 days prior to exposure to 25 μg mL^−1^ tunicamycin (*P* = 0.001). All experiments were performed three times and were considered significant for *P* < 0.05. See Table S3 (Supporting information) for additional data.

Differences in response of ER homeostasis regulators upon exposure to tunicamycin when *ifg‐1* was suppressed suggested that one or more may be required for improved survival. We tested the requirement for expression of *sca‐1* and *mdt‐15* for improved survival under ER stress induced by tunicamycin in the *ifg‐1*(*cxTi9279*) mutant. *sca‐1* RNAi abrogated enhanced survival in the *ifg‐1* mutant compared to *sca‐1* RNAi‐treated N2 wild‐type animals (Fig. 3A). Although this result indicated a requirement for *sca‐1*, it is interesting to note that *sca‐1* RNAi in the wild‐type background actually had a small but significant protective effect (Fig. 3A; Table S3, Supporting information). On the other hand, RNAi knockdown of *mdt‐15* did not abrogate improved survival in the *ifg‐1* mutant (Fig. 3B; Table S3, Supporting information). This result is consistent with recent evidence indicating that, while *mdt‐15* is essential for ER homeostasis through changes in the lipid synthesis profile of the ER, it does not alter proteostasis (Hou *et al*., 2014b). The fact that enhanced longevity through reduced *ifg‐1* expression is dependent on both *sca‐1* and *mdt‐15*, but here only required *sca‐1* for increased survival under ER stress, demonstrated that the dependence for lifespan and stress resistance phenotypes can be uncoupled.

Next, we tested the importance of the three ER stress sensor genes *atf‐6*,* pek‐1*, and *ire‐1* for enhanced survival upon exposure to tunicamycin in the *ifg‐1* mutant. Lowering *atf‐6* or *pek‐1* expression did not mitigate enhanced survival in the *ifg‐1* mutant (Fig. 3C,D; Table S3, Supporting information). Efficacy of RNAi for both genes was confirmed by testing for developmental effects in larval animals (not shown). In two of the three experiments performed, reducing *ire‐1* collapsed survival of the *ifg‐1* mutant to that of wild‐type animals on *ire‐1* RNAi (Fig. 3E; Table S3, Supporting information). Further testing showed that attenuation of the IRE‐1 substrate *xbp‐1* abolished enhanced ER stress resistance of the *ifg‐1* mutant in all tests (Fig. 3F; Table S3, Supporting information). Together, these data support an essential role for the IRE‐1 arm of the UPR^ER^ in mediating the protective effects exhibited in the *ifg‐1* mutant.

While unfolded proteins in the ER are known to activate the UPR^ER^, unfolded proteins in the cytoplasm trigger the HSR. To determine whether enhanced resistance to ER stress in *ifg‐1* animals was dependent on changes in the HSR governing cytoplasmic proteostasis, we tested the importance of the *hsf‐1* gene. Despite little or no dependence on survival under ER stress in wild‐type animals, results showed that RNAi targeting *hsf‐1* abolished the increased survival under ER stress in the *ifg‐1* mutant (Fig. 3G; Table S3, Supporting information). Analysis of total and translated transcript levels revealed that *ifg‐1* RNAi‐treated animals did not have significantly different expression of *hsf‐1* than control animals in the absence of stress (Fig. S3, Supporting information). However, *ifg‐1* RNAi led to preferential translation of *hsf‐1* subsequent to treatment with tunicamycin without increasing its transcription (Fig. S3, Supporting information). These results show that *ifg‐1* animals required *hsf‐1* for improved survival and had increased translation of *hsf‐1* under ER stress.

HSF‐1 and the FOXO transcription factor DAF‐16 are key regulators of proteostasis that are required for mediating longevity and stress resistance via the ILS pathway, as previously reviewed (Hartl *et al*., 2011). Based on results here that identified a requirement for *hsf‐1* for enhanced survival under ER stress when translation is attenuated, we analyzed the importance of *daf‐16* under the same conditions. Testing showed improved survival under tunicamycin stress in *ifg‐1* mutants compared to wild‐type animals when *daf‐16* was targeted via RNAi, although the protective effects were dampened (Fig. 3H; Table S3, Supporting information). This incomplete dependence on *daf‐16* was similar to its effect on lifespan in this background (Rogers *et al*., 2011).

We also tested the effects of genetically inhibiting translation at other points in initiation known to increase lifespan. RNAi targeting *ife‐2* (cap‐binding subunit of eIF4E)*, rps‐15* (small ribosomal subunit S15), or *iftb‐1* (beta subunit of *eIF2*, a component of the ternary complex representing another regulatory control point for translation initiation) increased survival in the presence of tunicamycin (Fig. S4; Table S4, Supporting information). These results suggested that enhanced survival under ER stress might be a general response of translation attenuation. We then treated animals with the chemical translation elongation inhibitor, cycloheximide, prior to exposure to tunicamycin, using a concentration of cycloheximide we found slowed growth in developing larvae and suppressed global translation in polysome profiling, phenotypes we observed with RNAi of the translation factor genes above (data not shown). Cycloheximide did not significantly impact median survival under ER stress conditions in either wild‐type or the *ifg‐1* mutant strain (Fig. 3I; Table S3, Supporting information), which suggested that inhibiting translation, per se, is not sufficient to increase survival under ER stress. Thus, targeting translation initiation through conserved regulatory nodes (i.e. the cap‐dependent/independent complex and ternary complex) might have promoted survival under ER stress in ways that extend beyond global reduction of translation.

### Reducing *ifg‐1* enhanced thermotolerance and resulted in higher constitutive and induced expression of genes encoding heat‐shock factors

**Figure 4:**
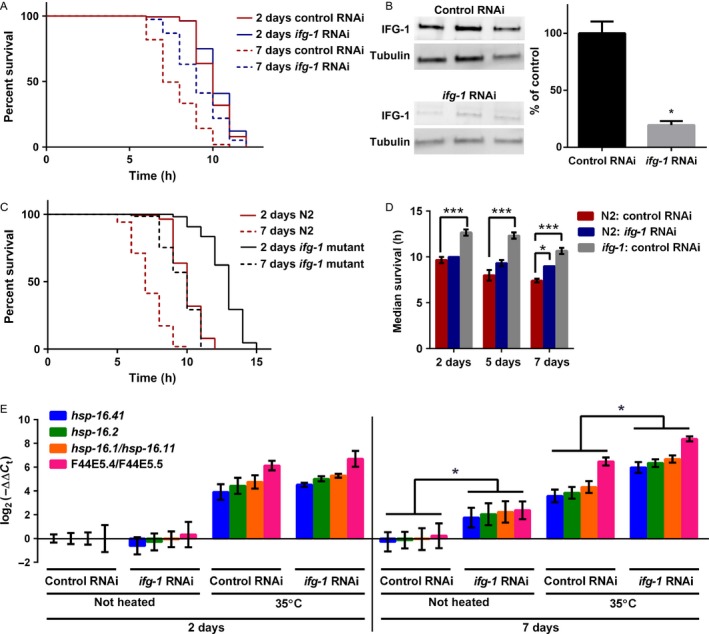
Enhanced thermotolerance was delayed compared with translation attenuation when *ifg‐1* was attenuated with RNAi and corresponded with upregulation of heat‐shock protein gene expression. (A) *ifg‐1* was inhibited for 2 or 7 days via RNAi beginning at adulthood in wild‐type N2 animals prior to incubation at 35 °C (i.e. heat stress conditions). Experiments were performed three times with similar results (see Table S5, Supporting information for additional data). Kaplan–Meier survival curves were compared using Mantel–Cox log‐rank test. Survival curve comparison was not significantly different after 2 days on RNAi for *ifg‐1* RNAi compared to the control (*P* = 0.06) but was highly significant for enhanced thermotolerance after 7 days (*P* < 0.0001). (B) Protein expression of IFG‐1 was measured after 2 days of *ifg‐1* RNAi treatment. Western blot quantification was from three independent experiments in which the average difference in IFG‐1 protein was standardized by the level of tubulin for each experiment. (*P < 0.05; Wilcoxon test; error bars indicate SEM). (C) Similar to (A), but for *ifg‐1*(*cxTi9279*) compared to N2. Experiments were performed three times with similar results (see Table S5, Supporting information for additional data). Kaplan–Meier survival curves were compared using Mantel–Cox log‐rank test. Results showed increased thermotolerance in the mutant compared to N2 for both time points (*P* < 0.0001 for each comparison). (D) Average median survival for three independent experiments corresponding to strains and conditions from (A) and (C) (**P < 0.001, ***P < 0.0001; ANOVA with post hoc Tukey test; error bars indicate SEM). (E) Expression of HSF‐1 target genes for the conditions and time points shown (*P < 0.05; ANOVA with post hoc Tukey test; error bars indicate SEM). All experiments were performed three times and were considered significant for *P* < 0.05.

Given the dependence on *hsf‐1* for the protective role of reducing *ifg‐1* under conditions that elicit an ER stress response, we analyzed differences in survival under heat stress that activates the cytoplasmic HSR. In two previous studies that tested thermotolerance in response to *ifg‐1* RNAi in adult *C. elegans*, one study found no protection from thermal stress after 2 days on *ifg‐1* RNAi (Pan *et al*., 2007), while the other showed significant protection 4 days after exposure to RNAi (Hansen *et al*., 2007). We re‐examined survival under continuous thermal stress (35 °C) after 2, 5, or 7 days of *ifg‐1* RNAi. Day one adults on *ifg‐1* RNAi for 2 days showed no enhanced protection from thermal stress (Fig. 4A; Table S5, Supporting information). However, longer exposure to *ifg‐1* RNAi resulted in increased thermotolerance (Fig. 4A,D; Table S5, Supporting information). Results here support both studies based on differences in the duration of dsRNA feeding prior to heat stress. To see whether the delay in enhanced thermotolerance was an artifact of the kinetics of RNAi and/or slow turnover of IFG‐1 protein, Western blot analysis was performed after 2 days on RNAi and showed that IFG‐1 was reduced by 80% (*P* < 0.05, Wilcoxon test; Fig. 4B). This suggested that slow turnover of IFG‐1 did not account for lack of a protective effect observed by that time. We also tested stress tolerance in the *ifg‐1*(*cxTi9279*) mutant, in which longevity and developmental phenotypes are less severe than *ifg‐1* RNAi‐treated animals. Results indicated markedly enhanced thermotolerance compared to wild‐type at both 2 and 7 days of adulthood (Fig. 4C,D; Table S5, Supporting information). Thus, although we cannot completely rule out that the delay may be due to a requirement for even lower levels of IFG‐1, the evidence collected does not support this interpretation.

To understand more about how lower IFG‐1 led to enhanced thermotolerance and the role of HSF‐1, we looked at induction of transcript levels for HSR genes after inhibiting expression of *ifg‐1* via RNAi for 2 or 7 days. We tested expression of *hsp‐16.1*/*hsp‐16.11*,* hsp‐16.2*,* hsp‐16.41*, and F44E5.4/F44E5.5 and found that after 2 days of *ifg‐1* RNAi, the induction of HSR genes under heat stress (35 °C) was comparable to control RNAi (Fig. 4E). However, after 7 days, animals on *ifg‐1* RNAi showed higher constitutive HSR gene expression compared with animals under control RNAi both before and after heating (Fig. 4E). We used the same time points to test expression of F44E5.4/F44E5.5 in animals treated with cycloheximide or RNAi for *ife‐2*,* rps‐15*, or *iftb‐1*. Both *rps‐15* and *iftb‐1* resulted in upregulation of this chaperone by day 7, similar to *ifg‐1* RNAi (Fig. S5, Supporting information). Interestingly, *rps‐15* and *iftb‐1* were also similar to *ifg‐1* in that they each are essential for translation and RNAi for these genes resulted in larval arrest in developing nematodes (data not shown). In contrast, *ife‐2*, which is required for cap‐dependent translation only, did not result in upregulation of heat‐shock gene expression (Fig. S5, Supporting information). It is also noteworthy that, as only one of five eIF4E orthologs in *C. elegans*,* ife‐2* suppression did not result in any developmental effects, even after multiple generations on RNAi (data not shown) and despite its protective effect in ER stress assays (Fig. S4A, Supporting information). To see whether the ability to inhibit development and reduce translation is required for the effect on heat‐shock gene expression, we tested the response to cycloheximide and found that this treatment, similar to *ife‐2*, failed to increase heat‐shock gene expression (Fig. S5, Supporting information). Thus, the kinetics of heat‐shock factor gene expression, while it potentially helped explain the delayed effect in enhancing thermotolerance under *ifg‐1* RNAi, also suggested potential differences in response to cap‐mediated vs. non‐cap‐mediated translational targeting.

### Genes essential for increased lifespan when *ifg‐1* is reduced were not required for enhanced thermotolerance

**Figure 5:**
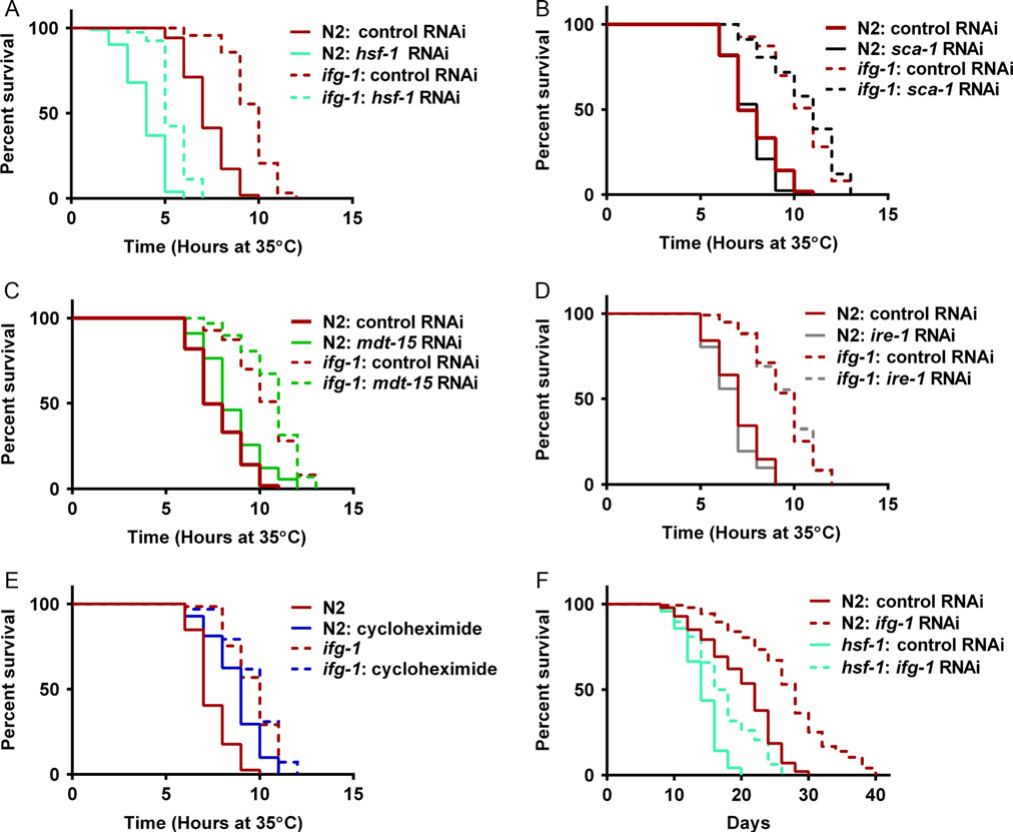
Enhanced thermotolerance by reducing *ifg‐1* did not require ER regulators and was not entirely dependent on *hsf‐1*. Kaplan–Meier survival curves in A–F were compared using Mantel–Cox log‐rank test. Survival at 35 °C was scored after 7 days under RNAi conditions indicated. Enhanced survival in *ifg‐1*(*cxTi9279*) compared to N2 was observed under the same RNAi treatment at *P* < 0.0001 for experiments in A–D. (E) Adults were pretreated with 0.5 mm cycloheximide for 7 days prior to scoring survival at 35 °C. Survival was enhanced in for cycloheximide‐treated N2 (*P* < 0.0001) but not for cycloheximide‐treated *ifg‐1*(*cxTi9279*) (*P* = 0.30). Despite lack of additional protection in *ifg‐1*(*cxTi9279*) treated with cycloheximide, survival was still enhanced over N2 under this condition (*P* < 0.0001). (F) Longevity of *hsf‐1*(*sy441*) mutants on *ifg‐1* RNAi was enhanced compared to mutants under control RNAi (*P* < 0.0001). All experiments were performed three or more times and were considered significant for *P* < 0.05. See Tables S6 and S7 (Supporting information) for additional data.

As demonstrated above, *hsf‐1* was required for enhanced survival in the *ifg‐1* mutant exposed to tunicamycin (Fig. 3G). To test whether *ifg‐1* required *hsf‐1* for enhanced thermotolerance, we treated wild‐type and *ifg‐1* mutant animals with *hsf‐1* RNAi for 7 days prior to heat stress. Although reducing *hsf‐1* severely decreased heat tolerance, the *ifg‐1* mutant demonstrated enhanced survival compared to N2 wild‐type animals (Fig. 5A; Table S6, Supporting information), indicating that there is a HSF‐1‐independent protective effect in the *ifg‐1* translation mutant. RNAi of neither *sca‐1*,* mdt‐15*, nor *ire‐1* had an effect on the ability of the *ifg‐1* mutant to enhance thermotolerance (Fig. 5B–D and Table S6, Supporting information). This further uncoupled survival phenotypes, demonstrating that enhanced thermotolerance does not require the same genes that are required for enhanced resistance to ER stress or lifespan. Once again, we tested the effects of cycloheximide as a chemical inhibitor of global translation. For this form of acute stress, thermotolerance was enhanced for wild‐type animals treated with cycloheximide for 7 days, unlike its lack of protective effect under tunicamycin stress. Interestingly, cycloheximide did not further enhance survival under thermal stress in the *ifg‐1* mutant (Fig. 5E; Table S6, Supporting information). This indicated that lowering translation, itself, had a protective effect to protein unfolding stress from heat.

Enhanced HSF‐1 activity indicated in Fig. 4E corresponded with enhanced survival when the UPR^ER^ was induced with tunicamycin. Although null mutants are not viable for *ifg‐1* or *hsf‐1* in *C. elegans*, we assessed the effect of *ifg‐1* RNAi on lifespan in a *hsf‐1*(*sy411*) reduced‐function background. Results indicate that *ifg‐1* RNAi was able to extend lifespan in this background (Fig. 5F and Table S7, Supporting information), in contrast to a different method of translation inhibition in another study that found RNAi knockdown of genes encoding ribosomal subunits, including *rps‐15*, failed to extend lifespan in the *hsf‐1*(*sy411*) background (Seo *et al*., 2013). Thus, although results in the current study indicated that *hsf‐1* was required for enhanced proteostasis elicited through attenuation of *ifg‐1* under ER stress, it was not required for increased lifespan. Conversely, regulators of ER homeostasis were not required for the enhanced cytoplasmic protective effects associated with lowering translation through *ifg‐1* prior to heat stress.

## Discussion

Reducing translation increases lifespan in model organisms and dysregulation is associated with age‐related diseases in humans, yet we do not fully understand how intracellular processes are altered to mediate these effects. By acting as a rate‐limiting modulator of protein synthesis, the cap‐binding complex subunit eIF4G is a nexus for translation‐mediated gene expression regulation. Previous studies suggest that eIF4G/IFG‐1 expression is regulated as an adaptive response to changing environmental conditions. In yeast, treatment with rapamycin or nutrient deprivation rapidly degrades eIF4G in a TOR‐dependent manner, but not the other cap‐binding complex subunits eIF4E and eIF4A (Berset *et al*., 1998). In *C. elegans*, where only one gene encoding eIF4G (*ifg‐1*) exists, adult animals fasted for 2 days show sharply diminished IFG‐1 protein expression (Rogers *et al*., 2011). In mammals, where there are three known forms of eIF4G, depletion of the eIF4GI form phenocopies nutrient deprivation and mTOR inhibition (Ramirez‐Valle *et al*., 2008). Furthermore, mRNAs important for growth and proliferation were preferentially excluded from polysomes (Ramirez‐Valle *et al*., 2008). Thus, lowering eIF4G may act as a ‘switch’ to lower and redirect energy expenditure when conditions are not conducive to growth and reproduction.

Here, we investigated how attenuation of this factor remodels intracellular processes and responses to perturbed protein homeostasis. We focused our investigation in the ER based on the observation that lowering *ifg‐1* shifts translation in favor of genes that encode components of the endomembrane system. We found that attenuating *ifg‐1* promoted survival under conditions that induce stress in the ER, the organelle responsible for maintaining the endomembrane system. A previous study showed that the preference for translation shifts from the cytosol to the ER when cap‐binding complex components (like eIF4G) become limiting (Lerner, 2006). This could explain why components of the endomembrane system are translationally preferred. The UPR^ER^ is induced in response to misfolded proteins and/or ER calcium imbalance. One of the key players in the enhanced ability to withstand ER stress and increase lifespan when *ifg‐1* is reduced is the ER calcium homeostasis regulator SCA‐1. This factor is highly expressed in contractile tissue (Zwaal *et al*., 2001) where it maintains a high level of calcium in the ER and helps ensure that calcium signaling in the cytoplasm is a transient event. We previously showed that translation and protein of SCA‐1 increases in response to lowering *ifg‐1* in *C. elegans* (Rogers *et al*., 2011). Its importance for enhanced stress tolerance and longevity suggest that calcium balance plays a major role in translation‐driven changes in proteostasis mediated by IFG‐1.

Another ER‐specific factor, IRE‐1 controls a major arm of the UPR^ER^. ER stress derepresses activation of this factor by freeing it of BiP (encoded in *C. elegans* by *hsp‐3* and *hsp‐4*). This, in turn, enables splicing of the retained intron in *xbp‐1* by IRE‐1 and production of the transcription factor XBP‐1. Although we did not observe increased activity of IRE‐1 when *ifg‐1* is attenuated according to the splicing status of *xbp‐1* under nonstressed conditions, we did observe a constitutive increase in overall abundance of *xbp‐1* in polysomes. Furthermore, we found that enhanced resistance to ER stress requires *ire‐1* and *xbp‐1*, suggesting that whatever role *hsf‐1* plays still requires canonical UPR^ER^ activation. Interestingly, a study investigating the effects of translation attenuation noted enhanced resistance to tunicamycin stress in yeast strains with ribosomal subunit deletions that *did not* require Hac1, the yeast ortholog of XBP‐1 (Steffen *et al*., 2012). However, the mechanism(s) underlying enhanced resistance to stress in those yeast mutants may be very different, as we do not know how or whether the stoichiometry of ribosomal subunits changes when *ifg‐1* is attenuated. For example, differential expression resulting from direct modulation of ribosomal constituents in yeast may be different from that elicited by reducing *ifg‐1* in *C. elegans*. In contrast to the protective effects of attenuating translation on ER stress outcomes in our model via *ifg‐1* in *C. elegans* and in the Steffen *et al*. model via ribosomal subunits in yeast, the translation inhibitor cycloheximide was not found to be protective under this condition, despite having a protective effect under thermal stress. Although this might suggest that global translation inhibition is not a major protective factor under the ER stress‐inducing conditions used here, it may also be due to a lack of ability to differentially regulate translation, offsetting normally protective effects of overall reduced translation. Additional studies are required to determine similarities and differences in altered translation among different models and methods.

Although finding that *hsf‐1* gene expression was required for enhanced survival under ER stress when *ifg‐1* is reduced was surprising, wild‐type animals showed no such requirement, suggesting that HSF‐1 transcriptional activity must be induced for the effect. In other systems, it was shown that ER stress responses neither automatically activate the HSR (mammalian tissue culture; Putics *et al*., 2008) nor require HSF‐1 for normal survival under ER stress‐inducing conditions (yeast; Hou *et al*., 2014a). However, constitutive activation of the HSR in yeast was able to partially compensate for loss of *ire1*, allowing cells to grow in the presence of ER stress (Liu & Chang, 2008). Some evidence for possible cross talk between *hsf‐1* and the ER is shown in the transcriptional induction of *sca‐1* to heat shock when *ifg‐1* is inhibited in the current study. Activating heat‐shock transcription factor in response to heat in other systems involves release from cytosolic HSP90, which keeps it sequestered in the cytoplasm prior to stress. Interestingly, we previously observed that *ifg‐1* RNAi diminished the translation and protein level of DAF‐21 (Rogers *et al*., 2011), the *C. elegans* ortholog of cytoplasmic HSP90. Although further investigation is required to determine exactly how boosting HSF‐1 enhances resistance to ER stress, results of the current study indicate a direct link between enhanced HSR function and amelioration of proteotoxicity in the ER.

In *C. elegans*, the ability to maintain proteostasis declines rapidly during the first day of adulthood (Ben‐Zvi *et al*., 2009; Labbadia & Morimoto, 2015). Results in the current study indicate that suppressing translation through *ifg‐1* in adult nematodes dramatically slows the decline in tolerance to heat normally observed in wild‐type animals. Another study showed that suppressing translation this way also enhances proteostasis in a polyglutamine model of protein folding disorder in nematodes (Kirstein‐Miles *et al*., 2013). In fact, numerous age‐related diseases are considered protein folding disorders resulting from a failure to maintain proteostasis, including Alzheimer's, Parkinson's, and Huntington's. In some instances, chronic activation of the UPR^ER^ is also associated with these disorders, as is dysregulation of calcium homeostasis (Thibault *et al*., 2007). Future studies are required to determine whether suppressing translation through eIF4G can potentially alleviate proteotoxicity in failing cellular systems to recover balance and health in affected tissues.

## Experimental procedures

### Nematode culture and strains

*Caenorhabditis elegans* strains were cultured at 20 °C and maintained on normal growth medium plates seeded with OP50 unless otherwise noted. Strains used included wild‐type N2, SJ4005 zcIs4 [*Phsp‐4::GFP; lin‐15*(*n765*)] V, *hsf‐1*(sy441), and *ifg‐1*(*cxTi9279*). All strains were permitted to grow at least three generations under normal, unstressed conditions prior to use in experiments.

### RNAi experiments

RNAi bacteria strains were cultured and utilized as previously described (Kamath *et al*., 2001). Induction was performed using 1 mm isopropyl‐β‐D‐thiogalactopyranoside (IPTG) in all cases. NGM agar plates prepared this way are referred to as ‘RNAi plates’. Bacteria expressing dsRNA included empty vector L4440 (Addgene, Cambridge, MA, U.S.A.), *ifg‐1* (M110.4), *sca‐1* (K11D9.2), *mdt‐15* (R12B2.5), *ire‐1*(C41C4.4), *atf‐6* (F45E6.2), *pek‐1* (F46C3.1), *xbp‐1* (R74.3), *hsf‐1* (Y53C10A.12), *daf‐16* (R13H8.1), *ife‐2* (R04A9.4)*, rps‐15* (F36A2.6)*,* and *iftb‐1* (K04G2.1) from the Ahringer library (Source BioScience, Nottingham, U.K.).

### Lifespan

Synchronized populations of nematodes were obtained from gravid adults treated with bleach to remove eggs. Eggs were permitted to hatch overnight in 10 mL of S Basal (0.1 m NaCl, 5.74 mm K_2_HPO_4_, 44.09 mm KH_2_PO_4_, 1 mL cholesterol (5 mg mL^−1^ in ethanol), in H_2_O to 1 L) before placing on plates spotted with OP50. We have also tested synchronization methods with timed egg‐lay or bleaching followed immediately by placing eggs on bacteria (a slightly less stringent form of synchronization) and obtained similar results. Upon the first day of adulthood, worms were transferred to RNAi plates that, in addition to 1 mM IPTG, contained 25 mg mL^−1^ carbenicillin and 50 μg mL^−1^ 5‐fluoro‐2′‐deoxyuridine (FUdR) to inhibit egg production. Lifespans were scored as day 0 from the time of first RNAi exposure. Nematodes were assessed for life every 2 days by gently tapping the worm with a platinum wire. Worms were scored as dead and removed from the plate upon failure to respond to several taps. Worms were censored if they died because of vulval rupture, internal hatching of progeny, or if they crawled off the plate. Rates of censorship were low in all experiments except those involving the *ifg‐1*(*cxTi9279*) mutant, in which a developmental defect results in frequent ruptures during adulthood (> 30%).

### Survival in response to tunicamycin treatment

Survival under tunicamycin stress was performed similar to a method previously described (Taylor & Dillin, 2013). Strains were synchronized by bleaching and eggs were allowed to grow on OP50. On the first day of adulthood worms were transferred to RNAi plates spotted with HT115 bacteria containing dsRNA as indicated and treated with FUdR as described above. After 2 days, worms were transferred to new RNAi plates containing a final concentration of 25 μg mL^−1^ tunicamycin dissolved in DMSO. These plates were spotted with bacteria 1 day prior to addition of tunicamycin, which was added 1 day prior to transferring worms. Control plates lacking tunicamycin contained an equivalent amount of DMSO. Survival was scored beginning at the time of tunicamycin exposure.

### Thermotolerance

Synchronized populations of worms were grown until adulthood on OP50 and maintained at 20 °C. Upon reaching day 1 of adulthood, nematodes were transferred to RNAi plates in the absence of FUdR and maintained at 20 °C. To prevent offspring contamination adults were moved to fresh RNAi plates every other day. Worms were shifted to 35 °C after 2, 5, or 7 days of exposure to RNAi and survival was scored by means of touch provocation. Worms not responding to touch were scored as dead.

### Chemical inhibition of translation

Cycloheximide (0.5 mm; Sigma, St. Louis, MO, U.S.A.) was added to RNAi plates containing bacteria bearing L4440 control vector or as otherwise indicated. Animals were exposed to cycloheximide 2 days prior to 25 μg mL^−1^ tunicamycin exposure or 7 days prior to shifting to 35 °C.

### Western blotting

Frozen worm pellets were ground in 8 m Urea, 2% SDS, 50 mm DTT, 50 mm Tris pH 7.4, with 1× Halt Protease Inhibitor Cocktail (Thermo Scientific, Waltham, MA, U.S.A.), allowed to solubilize on ice for 10 min, and debris was pelleted by centrifugation at 12 000 *g* for 3 min. Protein concentrations were determined using Qubit 2.0 protein assay (Life Sciences, Thermo Scientific, Waltham, MA, U.S.A.). Proteins were separated using 4–12% mini Protean TGX gels (Bio‐Rad) and transferred to PVDF membrane. Detection of IFG‐1 or phosphorylated eIF2α was performed using anti‐IFG‐1 polyclonal antibody (a kind gift from Brett Keiper; Contreras *et al*., 2008) or anti‐phospho‐eIF2α (Ser51; #9721; Cell Signaling Technology) along with goat anti‐rabbit Ig HRP‐conjugated secondary antibody (Thermo Scientific, Waltham, MA, U.S.A.). For IFG‐1 quantification, antibody against beta‐tubulin (Developmental Studies Hybridoma Bank) was used as a loading control with HRP‐conjugated goat anti‐mouse IgG (Thermo Scientific) used as a secondary antibody. Chemiluminescence was detected using the G: Box imager (Syngene, Cambridge, U.K.). For quantifying phosphorylated eIF2α, data were normalized using total protein quantified by loading into TGX stain‐free gels exposed to UV light for five min in a stratalinker prior to blotting. After transfer, the bands were visualized on a UV transilluminator with a fixed exposure time of 1 s. While all bands per lane were used for estimation of total protein loaded, a single representative band is shown in Fig. S1C (Supporting information). Background corrected band intensity was quantified using ImageJ.

### RNA isolation, cDNA synthesis, and qRT–PCR

RNA was isolated with TRIzol reagent (Invitrogen, Carlsbad, CA, USA) following the manufacturer's protocol for chloroform extraction. Samples were additionally processed with SurePrep RNA Cleanup and Concentration kit (Fisher BioReagents, Fair Lawn, NJ, USA). The polysome profile RNA isolation included 10 μg glycogen in each sample. A total of 200 ng RNA was reverse‐transcribed using QuantiTect Reverse Transcription kit (Qiagen, Valencia, CA, USA). qRT–PCR was performed in technical duplicate using KAPA SYBR FAST qPCR Master Mix on a LightCycler 480 (Roche Applied Science, Indianapolis, IN, USA). Target gene mRNA was normalized to the housekeeping gene *cdc‐42*, except for instances of polysome analysis, in which compared samples were normalized to the housekeeping gene *act‐1*, which we have found does not change among total and translated pools between N2 and *ifg‐1*, nor before or after exposure to tunicamycin. Changes in gene expression levels were analyzed by using the $2^{-\Delta \Delta C_{T}}$ method. Primer sequences are provided in Table S8 (Supporting information).

### Polysome profiling

Approximately 200 μL of whole worms were lysed on ice by grinding in solubilization buffer (ground in aliquots of 100 μL gently pelleted worms per 350 μL buffer; 300 mm NaCl, 50 mm Tris–HCL pH 8, 10 mm MgCl_2_, 1 mm EGTA, 200 μg mL^−1^ heparin, 400 U mL^−1^ RNAsin, 1 mm PMSF, 0.2 mg mL^−1^ cycloheximide, 1% Triton X‐100, 0.1% sodium deoxycholate). After grinding, an additional 350 μL solubilization buffer was added (1050 μL total) and the lysates were incubated on ice for 30 min. Lysates were centrifuged at 12 000 × *g* at 4 °C for 10 min to collect debris. Protein content of the lysate supernatants was estimated using Qubit protein assay (Thermo Scientific, Waltham, MA, U.S.A.) and loaded equally onto 10–50% sucrose gradients in high salt resolving buffer (140 mm NaCl, 25 mm Tris–HCL pH 8, 10 mm MgCl_2_). Gradients were resolved by ultracentrifugation in a Beckman SW41Ti rotor at 38 000 × *g* at 4 °C for 2 h. Fractions of the gradients were continuously monitored at absorbance of 254 nm using a Teledyne density gradient fractionator. The polysome fraction was collected and used for qRT–PCR as described above.

### GO enrichment analysis

Enrichment of biological GO terms among differentially translated genes was conducted using DAVID. A list of all genes in the original analysis was used for background correction. Broad GO functions were filtered out using the GO FAT option.

### Statistical analysis

All statistics were performed using GraphPad Prism 6 software (La Jolla, CA, U.S.A.). Two‐tailed *t*‐tests were performed on qRT–PCR samples to assess significance. Significance in instances of multiple comparisons was verified via ANOVA followed by post hoc Tukey test. Kaplan–Meier survival curves were plotted and compared using the Mantel–Cox log‐rank test. Western blots were analyzed using a Wilcoxon test.

## Author contributions

AH, JR, SS, SC, and ANR performed experiments. ANR and AH designed experiments and wrote the manuscript.

## Funding

Some strains were provided by the CGC, which is funded by NIH Office of Research Infrastructure Programs (P40 OD010440). This work was supported by grants from the National Institute on Aging of the National Institutes of Health (R00AG037621) and by The Ellison Medical Foundation (AG‐NS‐1087‐13). Research reported in this publication was also supported by an Institutional Development Award (IDeA) from the National Institute of General Medical Sciences of the National Institutes of Health under grant numbers P20GM0103423 and P20GM104318.

## Conflict of interest

The authors have no conflict of interests to declare.

## Supporting information


**Fig. S1** Impact of attenuating *ifg‐1* on ER stress sensors.

**Fig. S2** Reducing *ifg‐1* altered genes important for regulating normal ER function and stress responses.

**Fig. S3** Reducing *ifg‐1* altered *hsf‐1* translation under ER stress conditions.

**Fig. S4** Reducing translation longevity regulators *ife‐2, iftb‐1* or *rps‐15* promoted survival under ER stress.

**Fig. S5** Reduced *iftb‐1* or *rps‐15*, but not *ife‐2* or cycloheximide, constitutively activated expression of the HSR chaperone F44E5.4/F44E5.5 by day 7 of adulthood.

**Table S1** David GO term enrichment analysis of differentially translated genes showed five cellular component categories were altered after *ifg‐1* RNAi feeding.
**Table S2** Survival under 25 μg mL^−1^ tunicamycin‐induced UPR^ER^ stress was extended by attenuating *ifg‐1* expression.
**Table S3** Enhanced survival provided by reduced *ifg‐1* function under chronic UPR^ER^ stress (25 μg mL^−1^ tunicamycin) required certain proteostasis regulators.
**Table S4** Reduced expression of *ife‐2*,* rps‐15,* or *iftb‐1* promoted survival under ER stress (25 μg mL^−1^ tunicamycin).
**Table S5** Thermotolerance was enhanced with an extended period of translation attenuation through *ifg‐1* RNAi.
**Table S6** Thermotolerance in *ifg‐1*(*cxTi9279*) animals was only partially dependent on *hsf‐1*.
**Table S7** Lifespan was increased in *hsf‐1*(*sy441*) animals on *ifg‐1* RNAi.
**Table S8** Sequences of quantitative RT–PCR primers used in this study.
