# Supplementary figures and images for: Reducing translation through eIF4G/IFG‐1 improves survival under ER stress that depends on heat shock factor HSF‐1 in Caenorhabditis elegans
Source: Aging Cell. 2016 Aug 18;15(6):1027–38. doi: 10.1111/acel.12516 (PMC5114698; doi:10.1111/acel.12516)

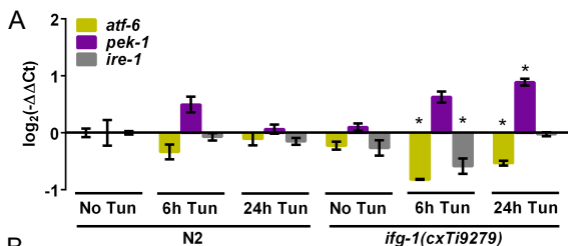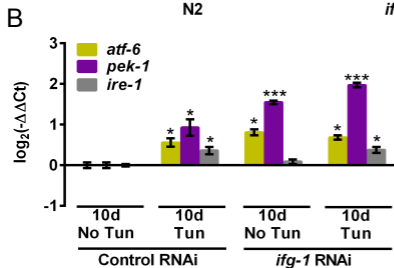

Supplement: Supplementary file 1 — Fig. S1 Impact of attenuating ifg‐1 on ER stress sensors. [file ACEL-15-1027-s001.pdf]

A

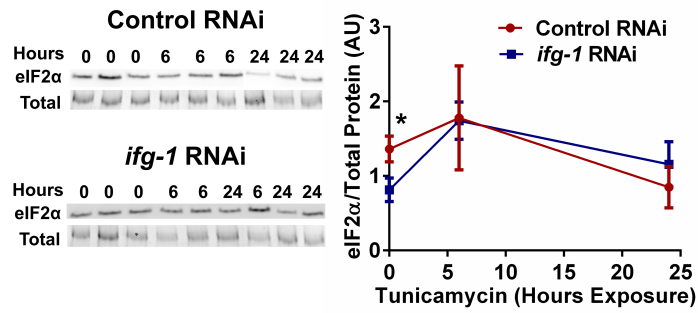

B

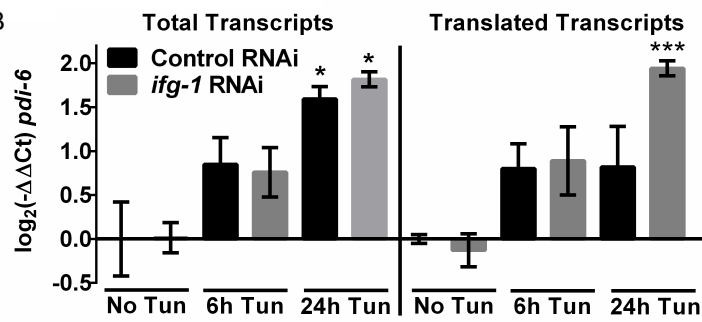

C

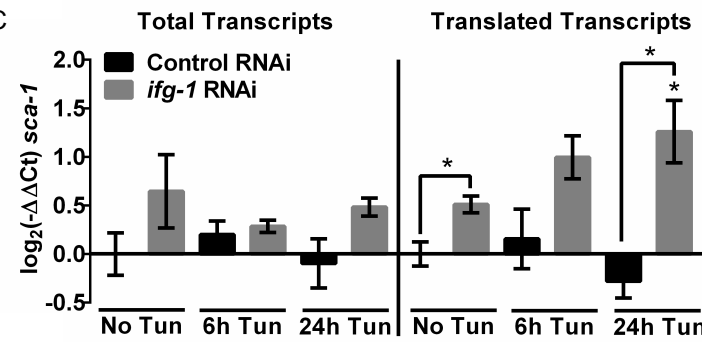

D

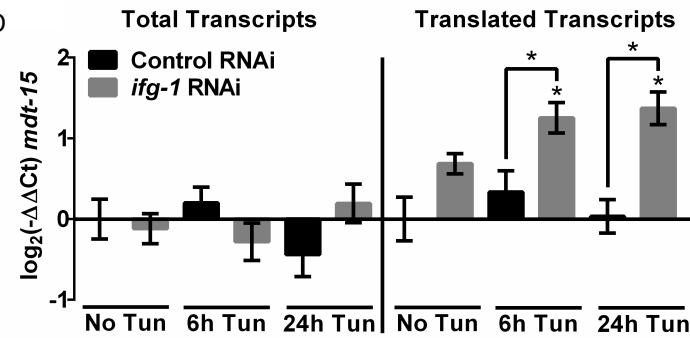

Supplement: Supplementary file 2 — Fig. S2 Reducing ifg‐1 altered genes important for regulating normal ER function and stress responses. [file ACEL-15-1027-s002.pdf]

## Total Transcripts

## Translated Transcripts

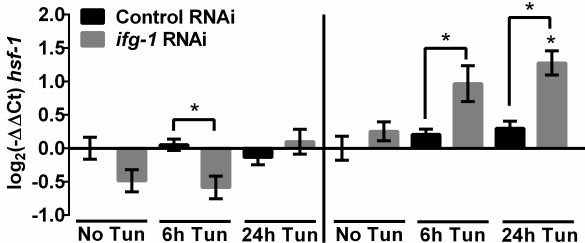

Supplement: Supplementary file 3 — Fig. S3 Reducing ifg‐1 altered hsf‐1 translation under ER stress conditions. [file ACEL-15-1027-s003.pdf]

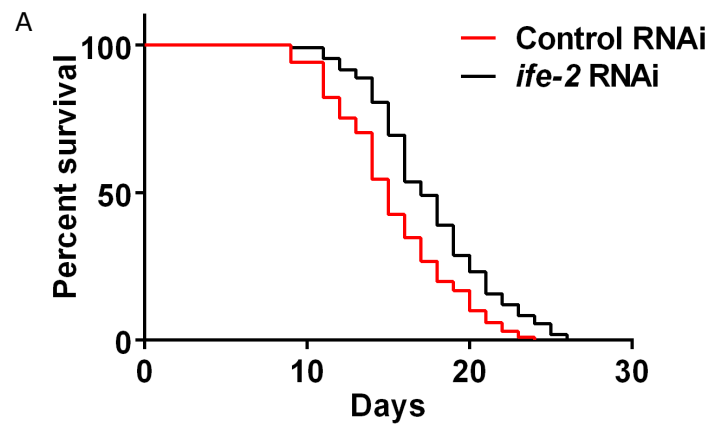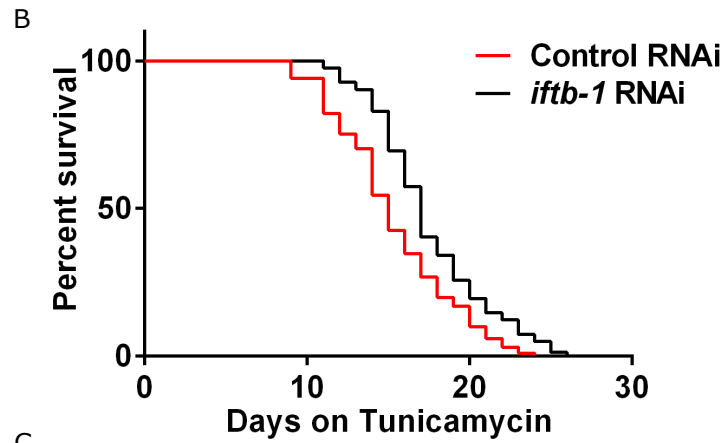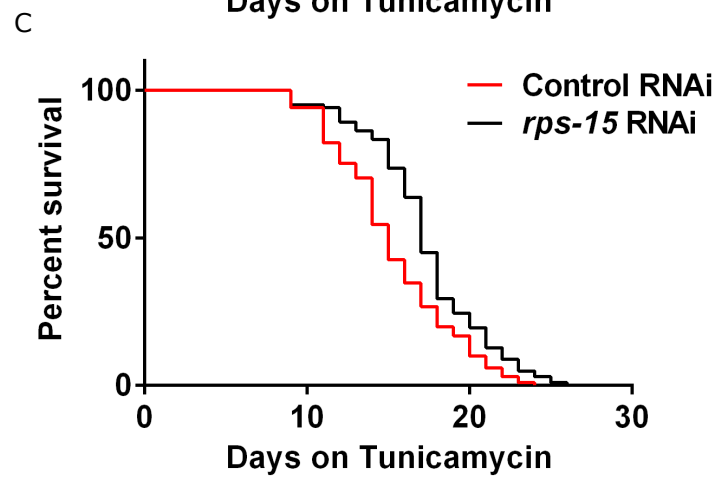

Supplement: Supplementary file 4 — Fig. S4 Reducing translation longevity regulators ife‐2, iftb‐1 or rps‐15 promoted survival under ER stress. [file ACEL-15-1027-s004.pdf]

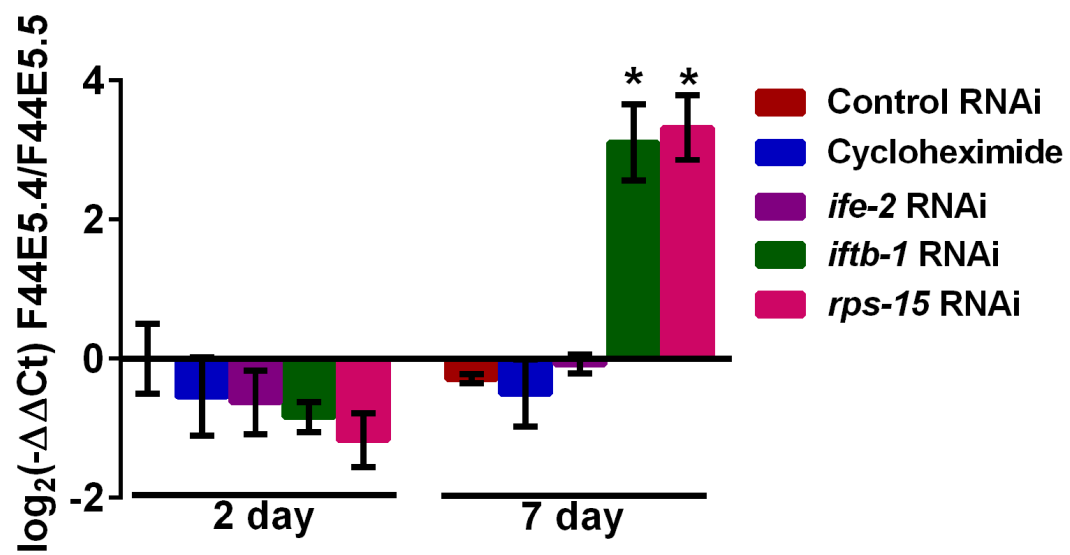

Supplement: Supplementary file 5 — Fig. S5 Reduced iftb‐1 or rps‐15, but not ife‐2 or cycloheximide, constitutively activated expression of the HSR chaperone F44E5.4/F44E5.5 by day 7 of adulthood. [file ACEL-15-1027-s005.pdf]
