# Supplementary material for: Reducing translation through eIF4G/IFG‐1 improves survival under ER stress that depends on heat shock factor HSF‐1 in Caenorhabditis elegans
Source: Aging Cell. 2016 Aug 18;15(6):1027–38. doi: 10.1111/acel.12516 (PMC5114698; doi:10.1111/acel.12516)
Supplement: Supplementary file 6 — Table S1 David GO term enrichment analysis of differentially translated genes showed five cellular component categories were altered after ifg‐1 RNAi feeding. Table S2 Survival under 25 μg mL−1 tunicamycin‐induced UPRER stress was extended by attenuating ifg‐1 expression. Table S3 Enhanced survival provided by reduced ifg‐1 function under chronic UPRER stress (25 μg mL−1 tunicamycin) required certain proteostasis regulators. Table S4 Reduced expression of ife‐2, rps‐15, or iftb‐1 promoted survival under ER stress (25 μg mL−1 tunicamycin). Table S5 Thermotolerance was enhanced with an extended period of translation attenuation through ifg‐1 RNAi. Table S6 Thermotolerance in ifg‐1(cxTi9279) animals was only partially dependent on hsf‐1. Table S7 Lifespan was increased in hsf‐1(sy441) animals on ifg‐1 RNAi. Table S8 Sequences of quantitative RT–PCR primers used in this study. [file ACEL-15-1027-s006.docx]

**SUPPORTING TABLES**

**Table S1. David GO term enrichment analysis of differentially translated genes indicated five cellular component categories were altered after *ifg-1* RNAi feeding.**

|  | **Cellular Component** | **Gene Name** | **Fold Enrichment** | **P Value** | **False Discovery Rate** |
| --- | --- | --- | --- | --- | --- |
| 1 | GO:0031410~cytoplasmic vesicle | *mel-11, copa-1, unc-11, sec-24.1, chc-1, cup-4, rme-6, sec-24.2, copb-2, ced-1, cope-1, sec-23* | 7.3 | 3.70E-07 | 0.000446 |
| 2 | GO:0031982~vesicle | *mel-11, copa-1, unc-11, sec-24.1, chc-1, cup-4, rme-6, sec-24.2, copb-2, ced-1, cope-1, sec-23* | 7.1 | 4.84E-07 | 0.000583 |
| 3 | GO:0012505~endomembrane system | C08H9.3, *npp-19, copa-1, imb-3, sec-24.2, sec-23, unc-11, sec-24.1, ostd-1, chc-1, cup-4, npp-19, npp-4, npp-10, cope-1, xpo-1, npp-17/rae-1* | 4 | 3.96E-06 | 0.004764 |
| 4 | GO:0044433~cytoplasmic vesicle part | *mel-11, copa-1, sec-24.1, chc-1, cup-4, copb-2, cope-1, sec-23* | 10.5 | 5.04E-06 | 0.006061 |
| 5 | GO:0016023~cytoplasmic membrane-bounded vesicle | *copa-1, unc-11, sec-24.1, chc-1, chc-1, cup-4, rme-6, sec-24.2, cope-1, sec-23* | 6.8 | 3.42E-05 | 0.041131 |

**Table S1.** Gene ontology analysis of differentially regulated genes after reducing *ifg-1* in N2 with RNAi. The top five gene ontology cellular component terms were listed with the fold enrichment and false discovery rate depicted in Fig. 1A.

**Table S2. Survival under 25 μg/mL tunicamycin-induced UPR^ER^ stress was extended by attenuating *ifg-1* expression.**

| **Exp** | **Tunicamycin** | **Strain** | **RNAi MS (days) N C** | **% MS** | **P Value** |
| --- | --- | --- | --- | --- | --- |
| 1^a^ | 25µg/mL  (30µM) | N2 | Control 14 96 0  *ifg-1* 18 99 0 | +28.6% | <0.0001 |
|  |  | *ifg-1* *(cxTi9279)* | Control 18 74 3 | +28.6% | <0.0001 |
| 2 | 25µg/mL  (30µM) | N2 | Control 16 87 0  *ifg-1* 18 79 0 | +12.5% | <0.0001 |
|  |  | *ifg-1* *(cxTi9279)* | Control 20 100 13 | +25% | <0.0001 |
| 3 | 25µg/mL  (30µM) | N2 | Control 14 161 0  *ifg-1* 16 148 0 | +14.3% | <0.0001 |
|  |  | *ifg-1* *(cxTi9279)* | Control 18 99 10 | +28.6% | <0.0001 |
| 4 | 25µg/mL  (30µM) | N2 | Control 13 168 0  *ifg-1* 15 160 0 | +15.4% | <0.0001 |
|  |  | *ifg-1* *(cxTi9279)* | Control 17 136 16 | +30.8% | <0.0001 |
| 5 | 50µg/mL  (60µM) | N2 | Control 16 111 0  *ifg-1* 18 77 0 | +12.5% | <0.0001 |

**Table S2.** Lifespans of wild-type (N2) and *ifg-1* *(cxTi9279)* loss-of-function animals subjected to tunicamycin stress 2 days after being fed control empty vector bacteria or bacteria expressing dsRNA targeting *ifg-1* beginning at adulthood. The experiment number is shown in the first column. P-values were considered significant for p < 0.05. Abbreviations are as follows: MS (median survival), N (observed number of deaths), C (number of censored animals), % MS (percent change in median survival compared to N2 control), P- values (log-rank Mantel-Cox test curve comparison).

^a^ Superscript denotes the experiment shown in Fig. 1B.

**Table S3. Enhanced survival provided by reduced *ifg-1* function under chronic UPR^ER^ stress (25 μg/mL tunicamycin) required certain proteostasis regulators.**

| **RNAi** | **Strain** | **Replicate** | **MS (days)** | **N** | **C** | **Avg MS (Days)** | **Avg MS for N2**  **Control**  **(P Value)** | **Avg MS for**  ***ifg-1(cxTi9279)***  **Control**  **(P Value)** |
| --- | --- | --- | --- | --- | --- | --- | --- | --- |
| Control (L4440) | N2 | 1^a^  2^b^  3^cdfgh^  4^e^  5^i^  6 | 13  14  12  12  13  14 | 168  161  84  79  67  141 | 0  0  1  0  1  0 | 13.0 ± 0.4 |  |  |
|  | *ifg-1* *(cxTi9279)* | 1^abc^  2^defg^  3^h^  4^i^  5  6  7  8 | 18  18  18  17  16  17  20  18 | 123  74  99  149  111  136  100  172 | 5  3  10  19  6  16  13  14 | 17.8 ± 0.4 | < 0.0001 |  |
| *atf-6* | N2 | 1^d^  2  3 | 10  13  14 | 125  135  140 | 0  1  1 | 12.3 ± 1.2 | 0.5060 | 0.0341 |
|  | *ifg-1* *(cxTi9279)* | 1^d^  2  3 | 18  18  18 | 93  90  161 | 11  19  17 | 18.0 ± 0 | < 0.0001 | 0.5630 |
| *daf-16* | N2 | 1^e^  2  3 | 10  11  10 | 134  142  165 | 0  0  0 | 10.3 ± 0.3 | 0.0024 | < 0.0001 |
|  | *ifg-1* *(cxTi9279)* | 1^e^  2  3 | 12  12  11 | 110  82  175 | 6  24  17 | 11.7 ± 0.3 | 0.0349 | < 0.0001 |
| *hsf-1* | N2 | 1^b^  2  3 | 12  9  10 | 127  127  82 | 0  0  0 | 10.3 ± 0.9 | 0.0116 | 0.0051 |
|  | *ifg-1* *(cxTi9279)* | 1^b^  2  3 | 12  14  11 | 99  115  189 | 3  3  1 | 12.3 ± 0.9 | 0.5400 | 0.0122 |
| *ire-1* | N2 | 1^h^  2  3 | 10  11  12 | 59  138  158 | 0  0  1 | 11.0 ± 0.6 | 0.0185 | 0.0005 |
|  | *ifg-1* *(cxTi9279)* | 1^h^  2  3 | 10  13  17 | 77  156  211 | 19  21  10 | 13.3 ± 2.0 | 0.8855 | 0.1564 |
| *mdt-15* | N2 | 1^c^  2  3 | 10  9  12 | 136  120  184 | 0  0  0 | 10.3 ± 0.9 | 0.0116 | 0.0051 |
|  | *ifg-1* *(cxTi9279)* | 1^c^  2  3 | 16  14  18 | 95  100  171 | 6  2  11 | 16.0 ± 1.2 | 0.1102 | 0.2645 |
| *pek-1* | N2 | 1^g^  2  3 | 14  12  14 | 121  172  164 | 0  0  0 | 13 ± 0.7 | 0.6438 | 0.0063 |
|  | *ifg-1* *(cxTi9279)* | 1^g^  2  3 | 16  16  18 | 97  105  185 | 8  5  9 | 16.7 ± 0.7 | 0.0140 | 0.2451 |
| *sca-1* | N2 | 1^a^  2  3 | 17  17  18 | 138  156  198 | 0  0  0 | 17.3 ± 0.3 | 0.0001 | 0.4553 |
|  | *ifg-1* *(cxTi9279)* | 1^a^  2  3 | 16  14  16 | 78  86  167 | 15  10  7 | 15.3 ± 0.7 | 0.0489 | 0.0413 |
| *xbp-1* | N2 | 1^f^  2  3 | 10  9  10 | 115  145  195 | 2  0  2 | 9.7 ± 0.3 | 0.0007 | < 0.0001 |
|  | *ifg-1* *(cxTi9279)* | 1^f^  2  3 | 10  10  9 | 86  68  189 | 16  36  15 | 9.7 ± 0.3 | 0.0005 | <0.0001 |
| *Control (L4440)*  *0.5mM Cycloheximide* | N2 | 1^i^  2  3 | 13  12  15 | 60  159  138 | 0  1  1 | 13.3 ± 0.9 | 0.6845 | 0.0211 |
|  | *ifg-1* *(cxTi9279)* | 1^i^  2  3 | 17  18  20 | 155  123  176 | 12  2  12 | 18.3 ± 0.9 | 0.0147 | 0.5922 |

**Table S3.** ER stress survival of wild-type N2 and *ifg-1* *(cxTi9279)* animals subjected to 25 μg/mL tunicamycin 2 days after being fed bacteria expressing dsRNA for the genes indicated beginning at adulthood. The experiment number is shown in the first column. P-values were considered significant for p < 0.05. Abbreviations are as follows: MS (median survival), N (observed number of deaths), C (number of censored animals), Avg MS (average median survival among replicates), Avg MS for N2 Control (p-value for average median survival of the test condition compared back to N2 on control RNAi), and Avg MS for *ifg-1(cxTi9279)* Control (p-value for average median survival of the test condition compared back to *ifg-1(cxTi9279)* on control RNAi). P-values were calculated from two-tailed unpaired t-tests with Welch’s correction of median survivals.

^a^Experiment shown in Fig. 3A. ^b^Experiment shown in Fig. 3G. ^c^ Experiment shown in Fig. 3B. ^d^ Experiment shown in Fig. 3C. ^e^ Experiment shown in Fig. 3H. ^f^ Experiment shown in Fig. 3F. ^g^ Experiment shown in Fig. 3D. ^h^ Experiment shown in Fig. 3E. ^I^ Experiment shown in Fig. 3I.

**Table S4. Reduced expression of *ife-2,* *rps-15* or *iftb-1* promoted survival under ER stress.**

| **RNAi** | **Strain** | **Replicate** | **MS (days)** | **N** | **C** | **Avg MS (Days)** | **Avg MS for Control N2**  **(P Value)** |
| --- | --- | --- | --- | --- | --- | --- | --- |
| Control (L4440) | N2 | 1  2^abc^  3 | 15  15  16 | 89  101  140 | 1  0  0 | 15.3 ± 0.3 |  |
| *ife-2* | N2 | 1  2^a^  3 | 17  17  18 | 101  108  135 | 0  1  3 | 17.3 ± 0.3 | 0.0132 |
| *iftb-1* | N2 | 1  2^b^  3 | 16  17  18 | 91  82  132 | 2  0  0 | 17.0 ± 0.6 | 0.0824 |
| *rps-15* | N2 | 1  2^c^  3 | 18  17  19 | 102  102  132 | 0  1  2 | 18.0 ± 0.6 | 0.0248 |

**Table S4.** ER stress survival of wild-type N2 animals subjected to 25 μg/mL tunicamycin following 2 days of RNAi for *ife-2*, *rps-15* or *iftb-1* beginning at day 1 of adulthood. The replicate number is shown in the third column. P-values are considered significant for p < 0.05. Abbreviations are as follows: MS (median survival), N (observed number of deaths), C (number of censored animals), Avg MS (average median survival of replicates), Avg MS for N2 control (p-value for average median survival of the test condition compared back to N2 on control RNAi), *P*-values (Two tailed unpaired t-test with Welch’s correction of median survivals).

^a^ Experiment shown in Fig. S4A.^b^ Experiment shown in Fig. S4B. ^c^ Experiment shown in Fig. S4C.

**Table S5. Thermotolerance was enhanced with an extended period of translation attenuation through *ifg-1* RNAi.**

| **Exp** | **Strain** | **Day** | **RNAi MS (Hours) N C** | **% MS** | **P Value** |
| --- | --- | --- | --- | --- | --- |
| 1^a^ | N2 | 2 days  7 days | Control 10 138 7  *ifg-1* 10 132 14  Control 7 104 2  *ifg-1* 9 114 26 | 0%  +28.57% | 0.0573  <0.0001 |
|  | *ifg-1* *(cxTi9279)* | 2 days  5 days  7 days | Control 13 109 13  Control 12 80 1  Control 10 65 13 | +30%  +42.86% | <0.0001  <0.0001 |
| 2 | N2 | 2 days  5 days  7 days | Control 9 36 0  *ifg-1* 10 40 10  Control 9 30 10  *ifg-1* 9 40 9  Control 7.5 50 5  *ifg-1* 9 40 16 | +11.11%  0%  +20% | 0.0460  0.8373  0.0072 |
|  | *ifg-1* *(cxTi9279)* | 2 days  5 days  7 days | Control 13 47 3  Control 12 38 29  Control 11 67 11 | +30%  +33.33%  +46.67% | <0.0001  <0.0001  <0.0001 |
| 3 | N2 | 2 days  5 days  7 days | Control 10 81 12  *ifg-1* 10 68 12  Control 7 51 8  *ifg-1* 10 61 10  Control 8 98 2  *ifg-1* 9 100 28 | 0%  +42.86%  +12.5% | 0.2975  <0.0001  <0.0001 |
|  | *ifg-1* *(cxTi9279)* | 2 days  5 days  7 days | Control 12 137 12  Control 13 78 7  Control 11 110 14 | +20%  +33.33%  +37.5% | <0.0001  <0.0001  <0.0001 |
| 4 | N2 | 7 days | Control 7 85 10 |  |  |
| 5 | N2 | 7 days | Control 7 105 8 |  |  |
| 6 | N2 | 7 days | Control 8 94 31 |  |  |
| 7 | N2 | 5 days | Control 8 82 44  *ifg-1* 9 74 58 | +12.5% | 0.9920 |

**Table S5.** Thermotolerance of wild-type N2 and *ifg-1(cxTi9279)* exposed to the RNAi indicated from day 1 of adulthood. After 2 days, 5 days, or 7 days plates were shifted to 35°C and animals were monitored hourly for survival. The experiment number is shown in the first column. P-values were determined using log-rank Mantel-Cox test and were considered significant for p < 0.05. Abbreviations are as follows: MS (median survival), N (observed number of deaths), C (number of censored animals), % MS (percent median survival compared to N2 on control RNAi). ^a^ Superscript denotes the experiments shown in Fig. 4A,C.

**Table S6. Thermotolerance in *ifg-1(cxTi9279)* animals was only partially dependent on *hsf-1*.**

| **RNAi** | **Strain** | **Replicate** | **MS (Hours)** | **N** | **C** | **Avg MS (Hours)** | **Avg MS for**  **N2**  **Control**  **(P Value)** | **Avg MS for**  ***ifg-1(cxTi9279)***  **Control**  **(P Value)** |
| --- | --- | --- | --- | --- | --- | --- | --- | --- |
| Control (L4440) | N2 | 1^a^  2^b^  3^cd^  4^e^  5  6  7  8  9  10  11 | 7  7  7  7  7.5  8  7  8  8  8  7 | 104  114  105  79  50  98  85  94  90  67  68 | 2  7  8  4  5  2  10  31  0  24  18 | 7.4 ± 0.1 |  |  |
|  | *ifg-1* *(cxTi9279)* | 1^a^  2^b^  3^cd^  4^e^  5  6  7  8  9 | 10  10  11  10  10  10  11  10  10 | 92  118  110  65  139  52  67  149  54 | 10  9  14  13  32  2  11  15  10 | 10.2 ± 0.1 | < 0.0001 |  |
| *hsf-1* | N2 | 1^a^  2  3 | 4  6  5 | 103  132  75 | 0  7  1 | 5.0 ± 0.6 | 0.0452 | 0.0086 |
|  | *ifg-1* *(cxTi9279)* | 1^a^  2  3 | 5  6  6 | 80  33  68 | 1  2  8 | 5.7 ± 0.3 | 0.0197 | 0.0015 |
| *ire-1* | N2 | 1^b^  2  3 | 7  8  8 | 102  68  59 | 13  42  41 | 7.7 ± 0.3 | 0.5335 | 0.0072 |
|  | *ifg-1* *(cxTi9279)* | 1^b^  2  3  4 | 10  9  10  9 | 123  129  114  42 | 17  20  31  23 | 9.5 ± 0.3 | 0.0017 | 0.0804 |
| *mdt-15* | N2 | 1^c^  2  3 | 8  9  8 | 89  53  89 | 0  0  9 | 8.3 ± 0.3 | 0.0897 | 0.0161 |
|  | *ifg-1* *(cxTi9279)* | 1^c^  2  3 | 11  11  10 | 98  74  124 | 7  2  3 | 10.7 ± 0.3 | 0.0036 | 0.3144 |
| *sca-1* | N2 | 1^d^  2  3 | 8  9  10 | 81  48  71 | 37  19  32 | 9.0 ± 0.6 | 0.1019 | 0.1615 |
|  | *ifg-1* *(cxTi9279)* | 1^d^  2  3 | 11  10  9 | 57  50  101 | 27  9  40 | 10.0 ± 0.6 | 0.0389 | 0.74123 |
| *Control (L4440)*  *0.25mM Cycloheximide* | N2 | 1  2  3 | 9  9  8 | 62  98  95 | 2  0  6 | 8.7 ± 0.3 | 0.0445 | 0.0265 |
| *Control (L4440)*  *0.5mM Cycloheximide* | N2 | 1^e^  2  3 | 9  9  7 | 112  92  99 | 0  0  1 | 8.3 ± 0.7 | 0.2981 | 0.0988 |
|  | *ifg-1* *(cxTi9279)* | 1^e^  2  3 | 10  10  11 | 97  68  91 | 7  4  8 | 10.3 ± 0.3 | 0.0049 | 0.7814 |

**Table S6.** Thermotolerance of wild-type N2 and *ifg-1(cxTi9279)* animals after 7 days of RNAi for the genes indicated or 7 days of pre-treatment with cycloheximide. The experiment number is shown in the third column. P-values were considered significant for p < 0.05. Abbreviations are as follows: MS (median survival), N (observed number of deaths), C (number of censored animals), Avg MS (average median survival among replicates), Avg MS fo N2 control (p-value for average median survival of the test condition compared back to N2 on control RNAi), and Avg MS for *ifg-1(cxTi9279)* Control (p-value for average median survival of the test condition compared back to *ifg-1(cxTi9279)* on control RNAi). P-values were calculated from two-tailed unpaired t-tests with Welch’s correction of median survivals.

^a^ Experiment shown in Fig. 5A. ^b^ Experiment shown in Fig. 5D. ^c^ Experiment shown in Fig. 5C. ^d^ Experiment shown in Fig. 5B. ^e^ Experiment shown in Fig. 5E.

**Table S7. Lifespan was increased in *hsf-1(sy441)* animals on *ifg-1* RNAi.**

| **Exp** | **Strain** | **RNAi MLS (days) N C** | **% MLS** | **P Value** |
| --- | --- | --- | --- | --- |
| 1^a^ | N2 | Control 22 140 0  *ifg-1* 28 143 4 | +27.27% | <0.0001 |
|  | *hsf-1 (sy441)* | Control 14 140 2  *ifg-1* 17 126 4 | -36.36%  -22.73% | <0.0001  <0.0001 |
| 2 | N2 | Control 25 127 3  *ifg-1* 35 105 7 | +40% | <0.0001 |
|  | *hsf-1 (sy441)* | Control 15 135 6  *ifg-1* 19 119 9 | -40%  -24% | <0.0001  <0.0001 |
| 3 | N2 | Control 27 125 6  *ifg-1* 30 114 1 | +11.11% | <0.0001 |
|  | *hsf-1 (sy441)* | Control 16 140 0  *ifg-1* 18 126 1 | -40.74%  -33.33% | <0.0001  <0.0001 |

**Table S7.** Lifespan of wild-type N2 and *hsf-1(sy441)* animals on *ifg-1* RNAi beginning at adulthood. The experiment number is shown in the first column. P-values were considered significant for p < 0.05. Abbreviations are as follows: MLS (median lifespan), N (observed number of deaths), C (number of censored animals), % MLS (percent change in median lifespan compared to N2 on control RNAi), P-values were calculated using log-rank Mantel-Cox test curve comparison with N2 control RNAi. ^a^ Experiment shown in Fig. 5F.

**Table S8. Sequences of quantitative RT-PCR primers used in this study.**

| **Gene** | **Primer sequence 5’ 🡪 3’** | | **Tm** |
| --- | --- | --- | --- |
| *atf-6* | Fwd | ACAGAAAGTAAAACCA | 54.16 |
|  | Rev | GTTTCCGGTCTTCAGTAG | 54.71 |
| *dnj-7* | Fwd | CAAGACGAAGAGAAACGA | 52.87 |
|  | Rev | GTGACCAAATCCTCCGTG | 56.35 |
| F44E5.4/ | Fwd | CAGAATGGAAAGGTTGAG | 53.73 |
| F44E5.5 | Rev | GAGCTGCTTGATCTTTTG | 52.79 |
| *hsf-1* | Fwd | ATGACTCCACTGTCCCAA | 56.28 |
|  | Rev | TTGCCGATTGCTTTCTCTT | 56.04 |
| *hsp-16.1/* | Fwd | TGAATCTTCTGAGATTGT | 48.98 |
| *hsp-16.11* | Rev | CTTTAATTCTTGTTCTCC | 48.82 |
| *hsp-16.2* | Fwd | CACTTTACCACTATTTCC | 50.06 |
|  | Rev | AACAATCTCAGAAGACTC | 50.97 |
| *hsp-16.41* | Fwd | CTCCGTTCTCCATATTCT | 52.31 |
|  | Rev | CATCATTTACAATCTCCCC | 53.63 |
| *hsp-4* | Fwd | GCCAACGATCAAGGAAACA | 55.65 |
|  | Rev | TAGAAACGCCCAATCAGAC | 55.44 |
| *ire-1* | Fwd | TTCTGCCCATTTGTTGCC | 56.83 |
|  | Rev | CTCATTTCTCCAGTTTCTC | 53.68 |
| *mdt-15* | Fwd | TATTCAGAGACTTGAGCC | 52.42 |
|  | Rev | CCTCATATATTCGTCCTTC | 52 |
| *pdi-6* | Fwd | ACAGACTACAACGGACAAA | 55.15 |
|  | Rev | TGCTGCTTCCTGATGATT | 55.19 |
| *pek-1* | Fwd | CAAAGTCCCAAAGCTAAG | 54.05 |
|  | Rev | CAATCGCAAGGTTTCATC | 53.75 |
| *xbp-1* | Fwd | GTTGCACCAGTTGTCGTC | 57.73 |
| (all isoforms) | Rev | GATTGTGTTGTTCTTCTTGG | 54.88 |
| *xbp-1s* | Fwd | CTTTGAATCAGCAGTGGG | 55.36 |
| (spliced) | Rev | GATTGTGTTGTTCTTCTTGG | 54.88 |
| *xbp-1u* | Fwd | GAATCAGCAGCATTCATT | 53.3 |
| (unspliced) | Rev | GATTGTGTTGTTCTTCTTGG | 54.88 |
| Housekeeping genes | | |  |
| *act-1* | Fwd | GCTGGACGTGATCTTACTGATTACC | 57.7 |
|  | Rev | GTAGCAGAGCTTCTCCTTGATGTC | 57.4 |
| *cdc-42* | Fwd | CTGCTGGACAGGAAGATTACG | 54.4 |
|  | Rev | CTCGGACATTCTCGAATGAAG | 52.4 |
