# Supplementary material for: Reducing translation through eIF4G/IFG‐1 improves survival under ER stress that depends on heat shock factor HSF‐1 in Caenorhabditis elegans
Source: Aging Cell. 2016 Aug 18;15(6):1027–38. doi: 10.1111/acel.12516 (PMC5114698; doi:10.1111/acel.12516)
Supplement: Supplementary file 7 [file ACEL-15-1027-s007.docx]

**Supplemental Figures**

**
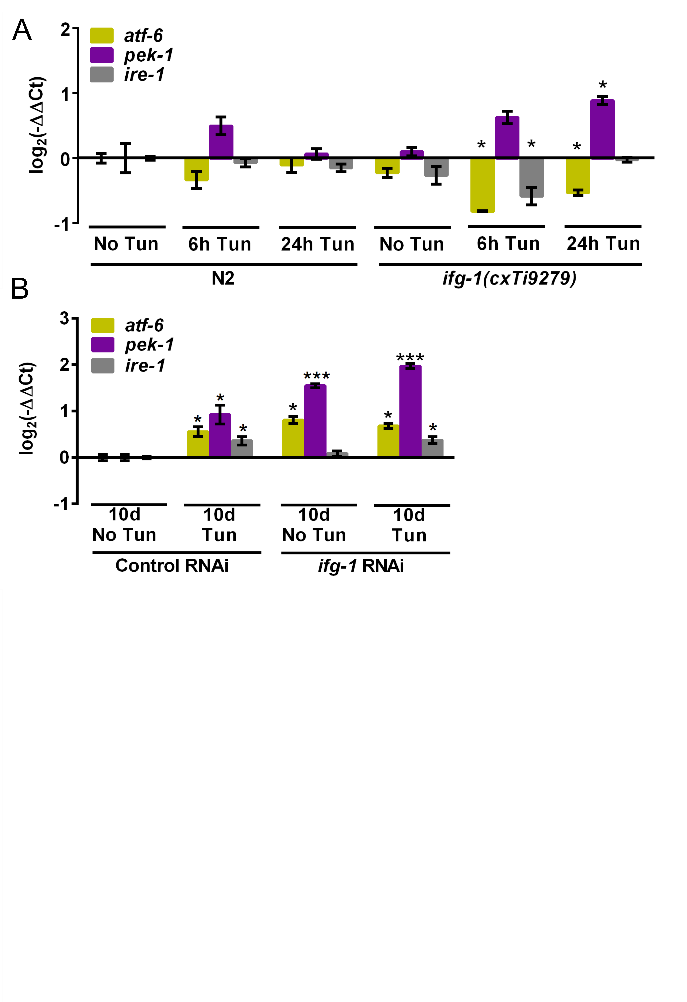
**

**Fig S1.** Impact of attenuating *ifg-1* on ER stress sensors. **A**) mRNA levels of *atf-6*, *pek-1*, and *ire-1* were measured using qRT-PCR in day 4 adult *ifg-1(cxTI9279)* and N2 after 6 or 24 hours of exposure to 25 μg/mL tunicamycin (6h Tun and 24h Tun, respectively). Samples were normalized to DMSO controls (No Tun). Results were from three separate experiments (* p < 0.05; two-tailed unpaired t-test; error bars represent SEM). **B**) N2 animals were exposed to 25 μg/mL tunicamycin after 2 days on *ifg-1* or control RNAi initiated at adulthood. mRNA levels of *atf-6*, *pek-1*, and *ire-1* were measured at day 10 of adulthood. Results were normalized to unexposed day 10 adults (* p < 0.05, *** p < 0.001, two-tailed unpaired t-test; error bars represent SEM). All experiments were performed three times and were considered significant for p < 0.05.

**
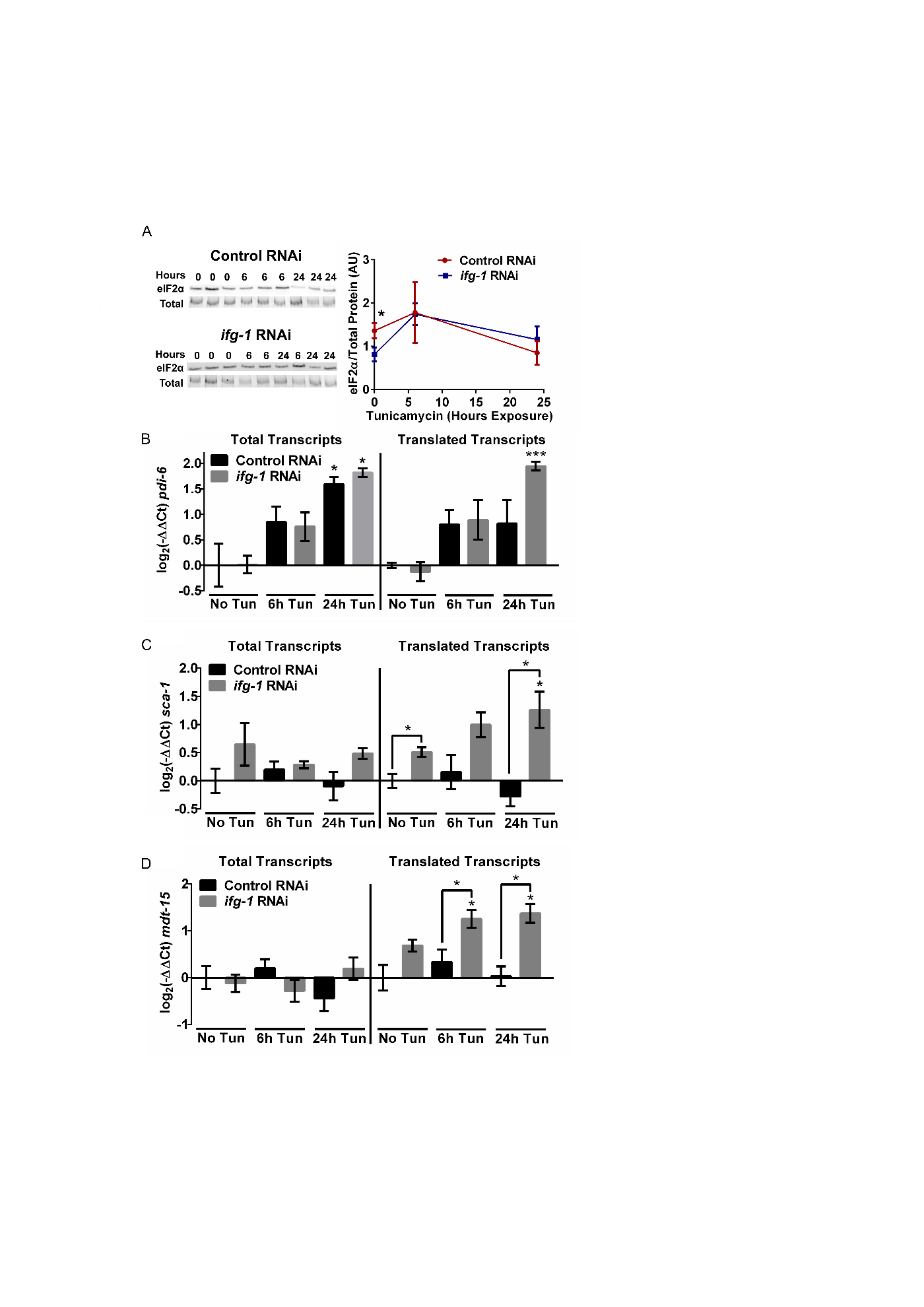
**

**Fig. S2** Reducing *ifg-1* altered genes important for regulating normal ER function and stress responses. N2 adults under *ifg-1* or control RNAi for 4 days were exposed to 25 μg/mL tunicamycin. **A**) Left panel shows Western blots for replicates of eIF2α phosphorylation assays after 6 or 24 hours. Results were standardized by tubulin levels and quantified in the right panel (*p < 0.05, two-tailed unpaired t-test; error bars indicate SEM). In **B-D**, total and polysome-associated (translated) transcript levels were determined for *pdi-6*, *sca-1*, and *mdt-15*, respectively (* p < 0.05; two-tailed t-test; error bars indicate SEM). Results were from four independent replicates. All expression values were normalized to DMSO-treated controls and were considered significant for p < 0.05.

**Fig. S3** Reducing *ifg-1* altered *hsf-1* translation under ER stress. Total and polysome-associated (translated) transcript levels for *hsf-1* were compared between *ifg-1* RNAi and control RNAi animals under no stress (No Tun) or after 6 and 24 hours of 25 μg/mL tunicamycin exposure (6h Tun and 24h Tun; * p < 0.05; two-tailed t-test; error bars indicate SEM). Results were from four separate experiments. All expression values were normalized to unstressed N2 controls and were considered significant for p < 0.05.

**
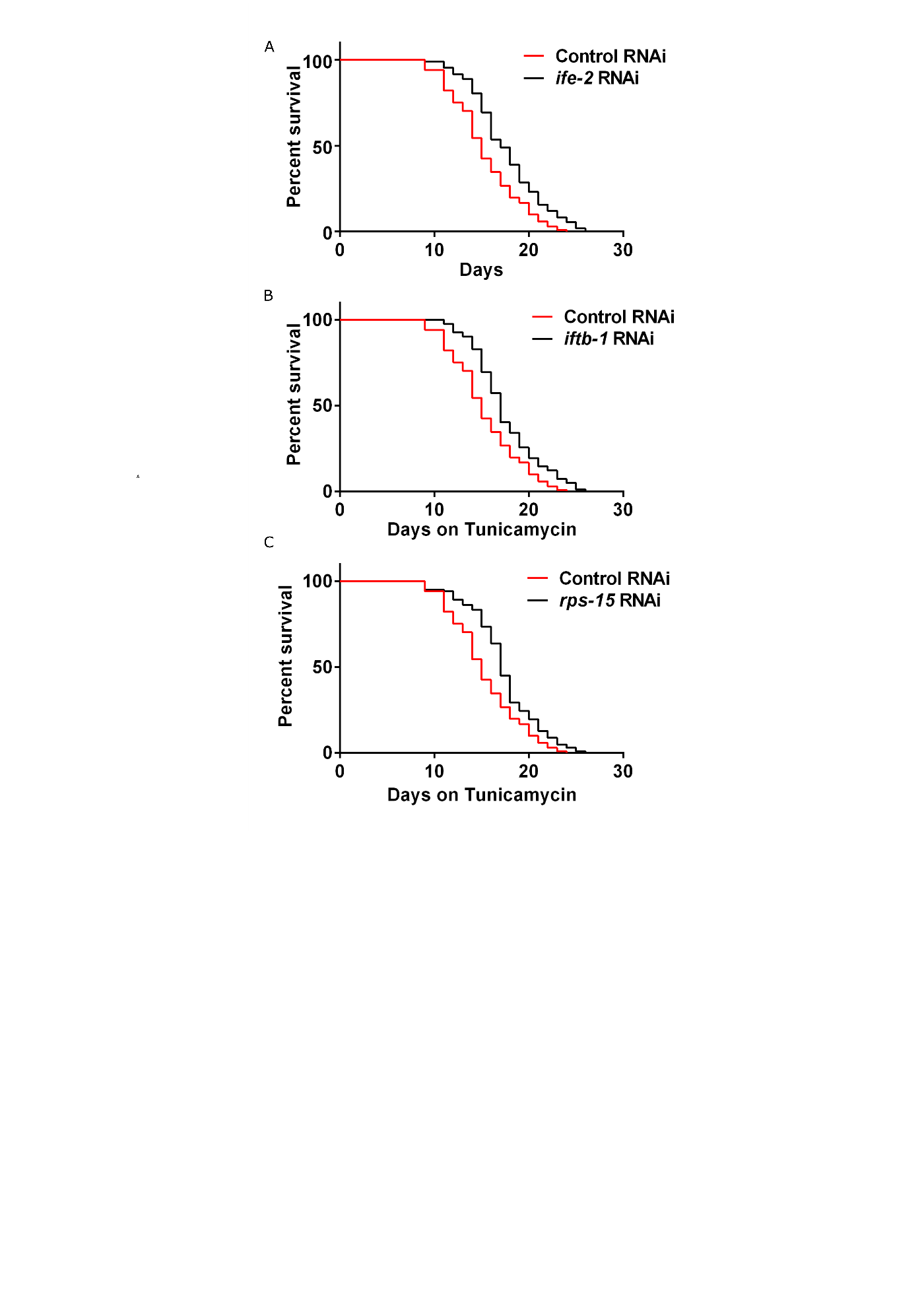
**

**Fig. S4** Reducing translation longevity regulators *ife-2*, *iftb-1* or *rps-15* promoted survival under ER stress. N2 animals were exposed to 25 μg/mL tunicamycin after 2 days on RNAi indicated. Kaplan-Meier survival curves were compared using Mantel-Cox log rank test. A) Survival was enhanced under *ife-2* RNAi compared to control (p < 0.0001). B) Survival was enhanced under *iftb-1* RNAi compared to control RNAi (p < 0.0001). C) Survival was enhanced under *rps-15* RNAi compared to control RNAi (p < 0.0001). Experiments were performed three times and were considered significant for p < 0.05. See Table S4 for additional data.

**
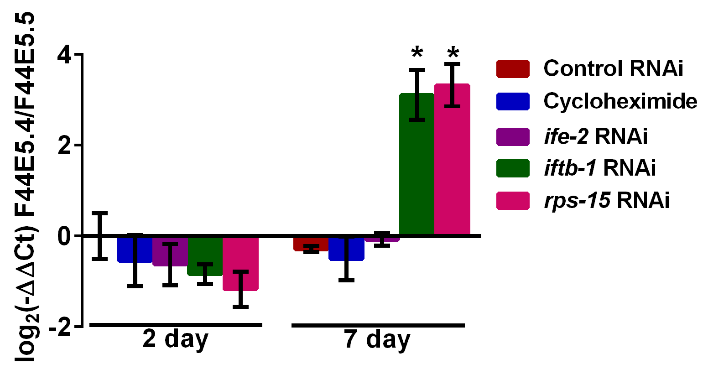
**

**Fig S5.** Reduced *iftb-1* or *rps-15*, but not *ife-2* or cycloheximide*,* constitutively activated expression of the HSR chaperone F44E5.4/F44E5.5 by day 7 of adulthood. N2 wild-type animals on RNAi shown or in the presence of 0.5mM cycloheximide on control RNAi from day 1 of adulthood were assayed for expression of F44E5.4/F44E5.5 after 2 or 7 days. All conditions were normalized to values for 2 days on control RNAi. (*p < 0.05, student’s two tailed unpaired t-test, error bars represent SEM). All experiments were performed three times and were considered significant for p < 0.05.
